# Short Total Synthesis of Ajoene

**DOI:** 10.1002/anie.201808605

**Published:** 2018-08-19

**Authors:** Filipa Silva, Shaista S. Khokhar, Danielle M. Williams, Robert Saunders, Gareth J. S. Evans, Michael Graz, Thomas Wirth

**Affiliations:** ^1^ School of Chemistry Cardiff University Park Place, Main Building Cardiff CF10 3AT UK; ^2^ Neem Biotech Roseheyworth Business Park North Abertillery NP13 1SX UK

**Keywords:** ajoene, allicin, garlic, organosulfur compounds, selenoxide elimination

## Abstract

We describe a short total synthesis of ajoene, a major biologically active constituent of garlic. The instability of allicin as the only other known alternative starting material has led to the development of a reliable procedure for the synthesis of ajoene from simple building blocks that is also suitable for upscale operations.

For a long time, garlic extracts and garlic‐based products have been used worldwide not only as food ingredients, but also as medicine for the prevention of stroke, coronary thrombosis, and atherosclerosis, as well as in the treatment of infections and vascular disorders.^1^ The therapeutic benefits of garlic are manifold and relate to the high concentrations of organosulfur compounds present in this plant. However, the instability of the major component allicin (**1**) limits the commercial viability of garlic extracts. Among other constituents of garlic, ajoene (**2**) derived from allicin is biologically active and more stable.^2^

To the best of our knowledge, there is only one reported synthesis of ajoene (**2**). Block and co‐workers described the biomimetic thermal rearrangement of allicin in aqueous acetone (Scheme 1),^3^ and recently this synthesis has been extended to produce a trifluorinated analogue.^4^ Although the synthesis is a one‐pot conversion, it suffers from low yields (34 %) owing to the formation reactive sulfur‐containing intermediates that also lead to side products. This reaction also does not allow for the synthesis of structurally modified or substituted analogues. More recently, Hunter and co‐workers reported a synthetic route to prepare a range of ajoene derivatives but this route could not be used to synthesize ajoene **2** itself.^5^

**Scheme 1:**
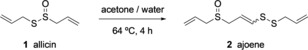
Block's synthesis of ajoene (**2**).

We present here an efficient total synthesis of ajoene (**2**). An isothiouronium salt was prepared by reaction of bromide **3** (R=OH) with thiourea, which was then hydrolyzed to the thiol and propargylated to form thioether **4**. The reaction of the hydroxy group in **4** with 2‐nitrophenyl selenocyanate and tributylphosphine produced the selenide **6 a** (Scheme 2). Alternatively, dibromide **3** (R=Br) can be treated with the phenylselenide anion generated in situ from diphenyl diselenide to afford bromide **5**. Compound **5** was then used to synthesize the propargylic thioether **6 b** using the same sequence of isothiouronium salt formation, hydrolysis, and propargylation. The overall yields for the reaction sequences to **6 a** and **6 b** are 29 % and 63 %, respectively. The selenium moiety will serve as the handle to introduce an alkene through a selenoxide elimination.

**Scheme 2:**
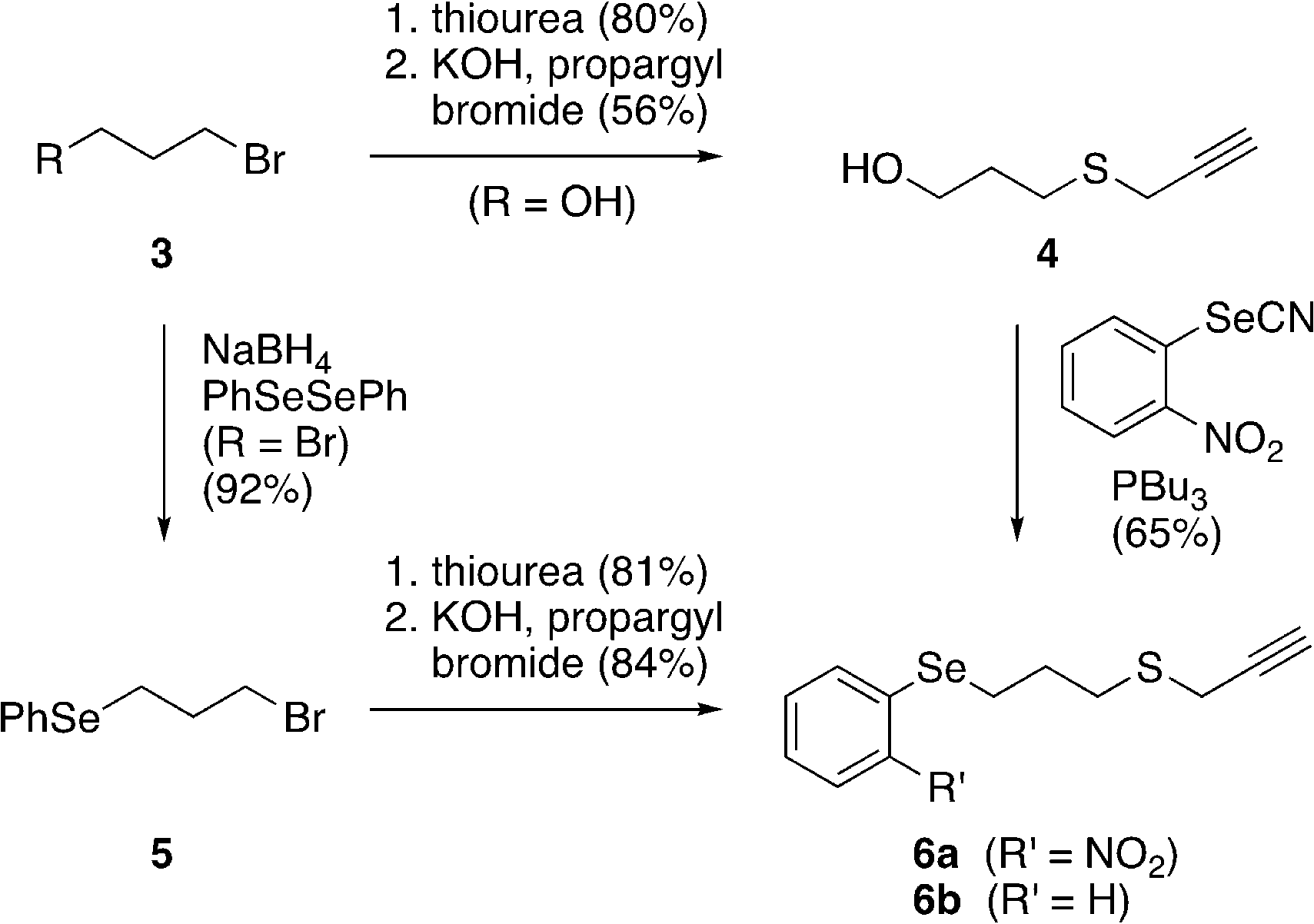
Synthesis of aryl propyl selenides **6**.

The next step of the synthesis involved the regioselective addition of thioacetic acid to the terminal alkyne **6**.^6^ The reaction was carried out by dissolving alkyne **6** in degassed toluene and heating to 85 °C with a radical initiator added to the solution, followed by the dropwise addition of thioacetic acid over 40 min using a syringe pump (Scheme 3). When ACCN [azobis(cyclohexanecarbonitrile)] was used as the radical initiator, compound **7 a** was obtained as a 2:3 mixture of the *E*/*Z* stereoisomers in 50 % yield (**7 b**: 2:3 *E*/*Z*, 64 %). For **7 b,** the yield was slightly improved to 71 % when AIBN [azobis(isobutyronitrile)] was used instead of ACCN. We could show that at this stage, the separation of the *E* and *Z* stereoisomers was possible by chromatography. However, the mixture was used in the next reaction as it is not stereospecific.

**Scheme 3:**
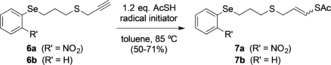
Radical addition of thioacetic acid to form derivatives **7**.

The hydrolysis of **7** to the thioenolate was achieved with potassium hydroxide in methanol, and the subsequent sulfenylation with thiosulfonic acid *S*‐alkyl ester **8** 7 occurred in good yields to give compound **9** (Scheme 4). The reaction was performed at −40 °C in order to avoid side reactions of the highly reactive thioenolate. The reaction with compound **7 a** (2:3 mixture of *E*/*Z* stereoisomers) afforded **9 a** in 73 % yield with the same *E*/*Z* ratio. When the reaction was performed with compound **7 b** (1:1 mixture of *E*/*Z* stereoisomers), compound **9 b** was obtained in 87 % yield and the ratio of *E*/*Z* stereoisomers changed to 2:3. As the stereoisomers could be separated by chromatography, a reaction with (*Z*)‐**7 b** was performed to determine whether isomerization to the *E* isomer occurs even at very low temperatures. Indeed, the reaction afforded **9 b** as a 3:2 mixture of *E*/*Z* stereoisomers in 85 % yield.

**Scheme 4:**
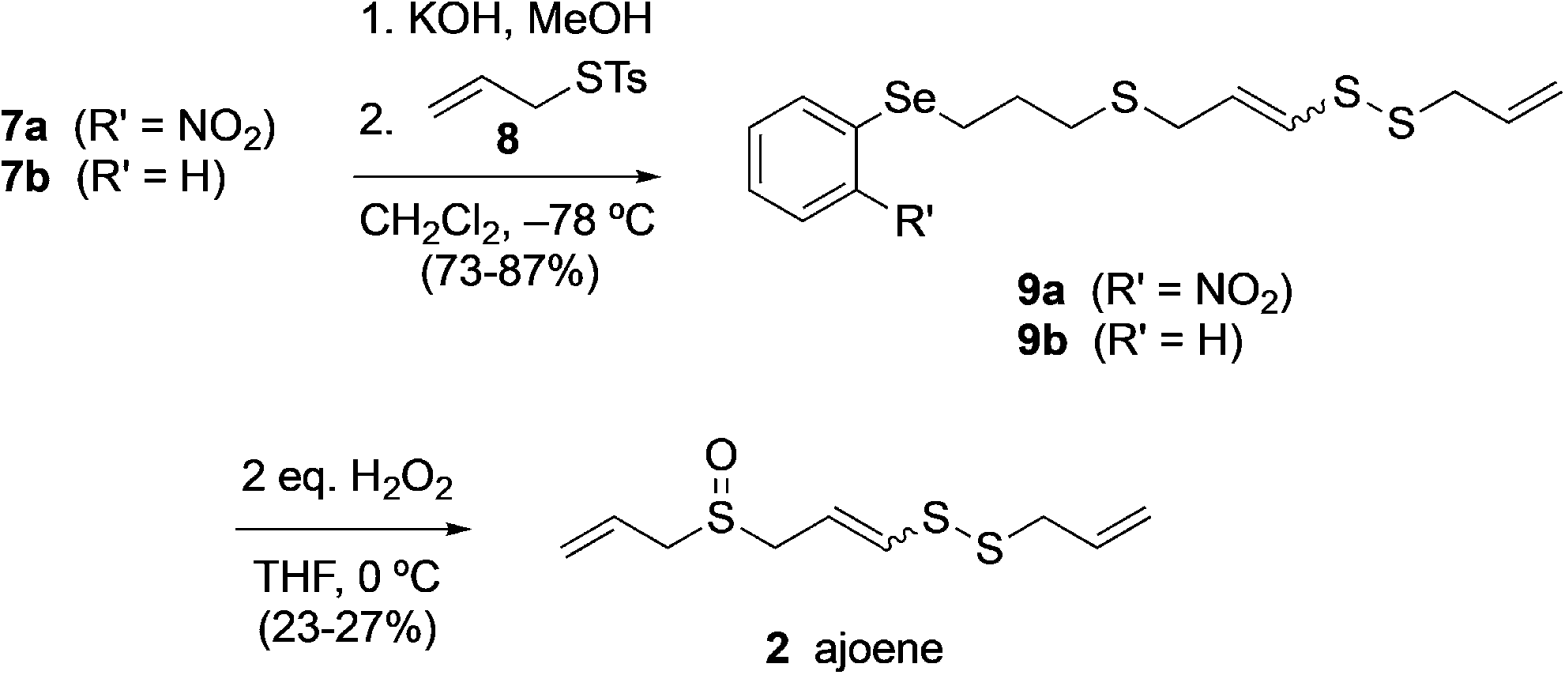
Synthesis of ajoene (**2**).

In the final step of the synthesis, compound **9** was treated with two equivalents of 30 % w/w hydrogen peroxide solution to form ajoene **2** in a 2:3 mixture of *E*/*Z* stereoisomers in 27 % (**9 a**) and 23 % (**9 b**) yield. The selenide as well as the sulfide functional group were oxidized and while the selenoxide undergoes a direct selenoxide elimination to form a double bond, the sulfoxide is retained in the product molecule. The *syn* elimination of alkyl aryl selenoxides is an efficient synthetic procedure to form alkenes. It is known that electron‐withdrawing substituents on the aromatic ring increase both the rate of elimination and the yield of the alkene.^8^ However, the use of the selenium derivative with an electron‐withdrawing substituent, **9 a**, did not show any advantage when compared with compound **9 b** as the yields were almost identical. A perselenenic acid byproduct might be able to catalyze the oxidation to the sulfoxide.^9^ The yields for the conversion from **9** into ajoene **2** were rather low on 0.3 mmol scale.

Further optimization studies of the selenoxide elimination and concomitant sulfur oxidation were carried out. For this study, compound **7 a** was used as the model substrate to find suitable reaction conditions. Product **10** can also be used as an ajoene precursor. Different oxidation conditions were investigated, and the results are presented in Table 1. The reaction of compound **7 a** with 2 equivalents of H_2_O_2_ (50 % w/w) afforded products **10** (23 %) and **11** (19 %; Table 1, entry 1). Increasing the amount of oxidant to 3 or 4 equivalents did not improve the yield of compound **10**, and compound **11** was still isolated (Table 1, entries 2 and 3). The complex urea hydrogen peroxide (UHP) was also used as an alternative to aqueous hydrogen peroxide solution. The reaction of compound **7 a** with 2 equivalents of UHP afforded, aside from compound **10** in 33 % yield, compound **11** in 17 % yield (Table 1, entry 4). Interestingly, when the reaction of compound **7 a** was carried out in the presence of NaIO_4_, compound **11** was isolated as the major compound in 50 % yield (Table 1, entry 5). *meta*‐Chloroperbenzoic acid (*m*CPBA) was also investigated as a suitable oxidant and found to give comparable yields (Table 1, entry 6).

**Table 1:** Optimization studies. 
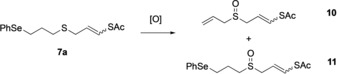

Entry	Oxidation conditions	Yield [%]		
		**7 a**	**10**	**11**
1	2 equiv H_2_O_2_ (50 % w/w), THF 0 °C (1 h)–rt (2 h)	20	23	19
2	3 equiv H_2_O_2_ (50 % w/w), THF 0 °C (1 h)–rt (2 h)	21	20	25
3	4 equiv H_2_O_2_ (50 % w/w), THF 0 °C (1 h)–rt (2 h)	–	12	9
4	2 equiv UHP, CH_2_Cl_2_ 0 °C (1 h)–rt (2 h)	6	35	6
5	2 equiv NaIO_4_, CH_3_OH/H_2_O 0 °C (2 h), then rt (6 h)	–	9	50
6	2 equiv *m‐*CPBA, CHCl_3_ 0 °C (1 h)–rt (2 h)	–	37	16
7	2 equiv H_2_O_2_ (50 % w/w), CH_2_Cl_2_ 1.5 equiv DIPA, 0 °C (1 h)–rt (2 h)	20	27	–
8	2 equiv *m*CPBA, CH_2_Cl_2_, 2 equiv DIPA 0 °C (1 h)–rt (2 h)	–	46	–
9	2 equiv *m*CPBA, CH_2_Cl_2_, 2 equiv DIPA 0 °C (1 h)–rt (24 h)	–	44	–

Independent of the reaction conditions, compounds **10** and **11** were the two major products formed and isolated. However, smaller amounts of other non‐identified side products were also detected. Areneselenenic acid generated during the selenoxide elimination is in equilibrium with its disproportionation products (diaryl diselenide and areneseleninic acid). Under neutral or acidic conditions, they can react with alkenes to generate side products. These side reactions can be suppressed by the addition of alkyl amines. When 1.5 equivalents of diisopropylamine (DIPA) were added to the reaction with H_2_O_2_, the formation of compound **11** was suppressed, but compound **10** was only isolated in 27 % yield. Adding 2 equivalents of DIPA to the reaction with *m*CPBA not only stopped the formation of compound **11**, but also improved the yield of product **10** to 46 % (Table 1, entry 8). Increasing the reaction time under the same reaction conditions did not affect the yield of compound **10** (Table 1, entry 9).

The complete synthesis of ajoene **2** was scaled up, with slightly different results being obtained.^10^ The synthesis of **5** proceeded with 58 % yield on 4 mol scale while the subsequent thiol formation and propargylation led to **6 b** in 87 % yield (2.9 mol). Radical addition of thioacetic acid proceeded similarly well compared to the small‐scale synthesis (**7 b**: 75 %, 1.4 mol) as did the thioacetate cleavage and thioallylation to **9 b** (74 %, 1.1 mol). The final oxidation to ajoene **2** displayed superior yields (65 %) compared to the small‐scale synthesis, and 169 g (0.72 mol) of ajoene **2** were isolated in about 90 % purity as determined by HPLC and NMR analysis.

Much of the research interest in ajoene **2** resides in its biological activity. It has been shown to have efficacy in a number of biological studies, including antithrombotic and antifungal activities.^11^ In order to further evaluate **2**, its activity in a biological assay was also examined. Ajoene's ability to act as a quorum sensing inhibitor (QSI) was selected as this is one of its more recent remarkable biological properties. Quorum sensing (QS) is a mechanism of cell–cell communication in bacteria facilitated by the secretion and detection of signaling molecules such as *N*‐acyl homoserine lactones in Gram‐negative bacteria.^12^ QS allows bacteria to synchronize specific gene expression, which has an impact on their pathogenicity and is thought to play a significant role in the formation of biofilms. Recent studies have shown that ajoene **2** is an effective QS inhibitor against *Pseudomonas aeruginosa* and *Staphylococcus aureus* and could be utilized for the treatment of chronic biofilm infections by exploiting the QS system.^13^ In this study, we employed a reporter strain (*Pseudomonas aeruginosa* Pa01 *lasB‐gfp*)13a whereby QS gene expression was monitored over time in response to ajoene treatment.

Two ajoene products were examined; **2 (synthetic)** as synthesized above and ajoene **2 (garlic)** extracted from garlic using the thermal rearrangement conditions.^3^ The results are expressed as a mixture of (*E*)‐ and (*Z*)‐ajoene. Both ajoene samples are effective QSIs as shown by their inhibition of the fluorescence in Figure 1, where a reduction in fluorescence is directly related to the downregulation of the QS gene *lasB*.

**Figure 1:**
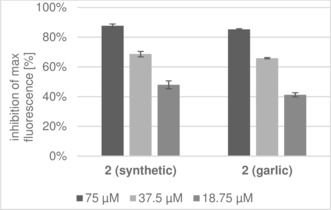
Inhibition of a *Pseudomonas aeruginosa* Pa01 *lasB‐gfp* reporter strain, where a decrease in fluorescence is directly related to QS‐controlled expression.

The samples show a very similar pattern of concentration‐dependent inhibition. This is reiterated in the IC_50_ calculations where ajoene **2 (garlic)** extracted from garlic had an IC_50_ value of 27.7 μm and synthetic ajoene **2 (synthetic)** had an IC_50_ value of 28.5 μm. The IC_50_ values are comparable between the different origins of ajoene **2**.

In conclusion, we have described an efficient total synthesis of ajoene from easily available starting materials. The simultaneous introduction of the allyl moiety and the sulfoxide group in the final step enabled the straightforward generation of the target molecule. Upscaling of the synthetic sequence was possible, leading to the synthesis of larger amounts of ajoene for the first time. Synthetic ajoene and ajoene derived from garlic were investigated regarding their efficiency as quorum sensing inhibitors.

## Experimental Section

Vinyl disulfide **9 a** (0.140 g, 0.33 mmol) was dissolved in THF (3 mL) and cooled to 0 °C under N_2_, and H_2_O_2_ (30 % w/w in H_2_O, 0.075 mL, 0.66 mmol) was added dropwise. The mixture was stirred for 1 h at 0 °C and then warmed to room temperature (2 h). Sat. aq. NaHCO_3_ (5 mL) was added, and the residue was extracted with EtOAc (2×10 mL). The combined organic fractions were washed with brine (2×10 mL) and dried over MgSO_4_. The solvent was removed under vacuum, and the resulting residue was purified by column chromatography to afford ajoene **2** (21 mg, 27 %, *E*/*Z=*1:1.8) as a pale‐yellow oil.

## Conflict of interest

The authors declare no conflict of interest.

## Supporting information

As a service to our authors and readers, this journal provides supporting information supplied by the authors. Such materials are peer reviewed and may be re‐organized for online delivery, but are not copy‐edited or typeset. Technical support issues arising from supporting information (other than missing files) should be addressed to the authors.

Supplementary
