# Supplementary material for: Short Total Synthesis of Ajoene
Source: Angew Chem Int Ed Engl. 2018 Aug 19;57(38):12290–3. doi: 10.1002/anie.201808605 (PMC6221122; doi:10.1002/anie.201808605)

Angewandte Chemie
Eine Zeitschrift der Gesellschaft Deutscher Chemiker

Supporting Information

Short Total Synthesis of Ajoene

*Filipa Silva, Shaista S. Khokhar, Danielle M. Williams, Robert Saunders, Gareth J. S. Evans, Michael Graz, and Thomas Wirth**

anie_201808605_sm_miscellaneous_information.pdf

Supporting Information

Contents

1. General considerations S2
2. General Scheme S3
3. Experimental part S4
4. Gene reporter assays to determine QSI effect S12
5. References S13
6. NMR spectra S14

1. General considerations

The reactions were performed using standard laboratory equipment. Air sensitive reactions were carried out under argon or nitrogen atmosphere using oven-dried glassware. Reactions were stirred using magnetic stirring and heated to specified temperatures using hotplates with temperature probe control and an adapted heating block. Lower temperatures were obtained using ice/water (0 ºC), dry ice/acetonitrile (–40 ºC), dry ice/acetone (–78 ºC). Büchi B-461, B-481 or B-490 were used for solvent evaporations (reduced pressure up to 15 mbar) and high vacuum apparatus was used to further dry the products. All chemicals were purchased from Sigma Aldrich, Alfa Aesar, Fisher Scientic, TCI UK, Fluorochem and used without further purification. Dry solvents were obtained from an MBRAUN SPS-800 solvent purification system.

All the reactions were monitored by thin-layer chromatography (TLC), which was performed on Merck Silica gel 60 F254 (0.20 m) and visualised by UV radiation (254 nm) or/and by staining with potassium permanganate solution (1.5 g $KMnO_4$, 10 g K_2CO_3, 1.25 mL 10% NaOH, 200 mL distilled H_2O). Manual column chromatography was performed using silica gel 60 (Merck, 230-400 mesh) under increased pressure (Flash Chromatography) or as gravitational column chromatography. The solvents used for the purification are indicated in the text and were purchased from fisher Scientific as laboratory grade. Automated column chromatography was performed on a Biotage® Isolera Four using Biotage® cartridges SNAP Ultra 10g, SNAP Ultra 25g, SNAP Ultra 50g, SNAP Ultra 100g. The solvents used for the purification are indicated in the text and were purchased from fisher Scientific as laboratory grade.

^{1}H NMR and ^{13}C NMR spectra were measured on Burker DPX 500 (500 MHz), Bruker DPX 400 (400 MHz), Bruker DPX 300 (300 MHz) instruments. The chemical shifts δ are given in ppm downfield of tetramethylsilane (δ = 0 ppm). Compounds or crude reaction mixtures were dissolved in either deuterated chloroform, deuterated methanol or deuterated acetonitrile. Coupling constants (*J*) are given in Hertz. The multiplicity of signals is designated: s = singulet, d = doublet, t = triplet, q = quartet, quin= quintet, dt = double of triplet, m = multiplet. Residual solvent peaks are 7.26 ppm for chloroform, 3.31 ppm for methanol and 1.94 ppm for acetonitrile. The molecular ions peaks values quoted for either molecular ion $[M]^+$, molecular ion plus hydrogen $[M+H]^+$, molecular uon plus sodium $[M+Na]^+$ or molecular ion plus $[M+K]^+$.

IR spectra were recorded on Shimadzu IR Affinity-1S apparatus. Wavenumbers are quoted in cm^{-1}. All compounds were measured neat directly on the crystal of the IR instrument. Melting points were measured using a Gallenkamp variable heater with samples in open capillary tubes.

2. General Scheme

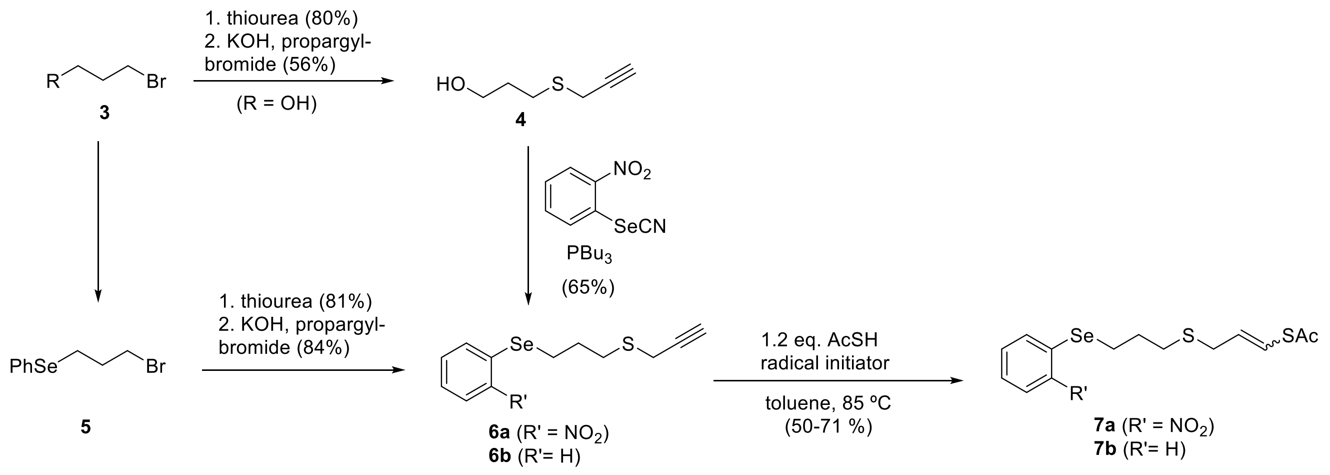

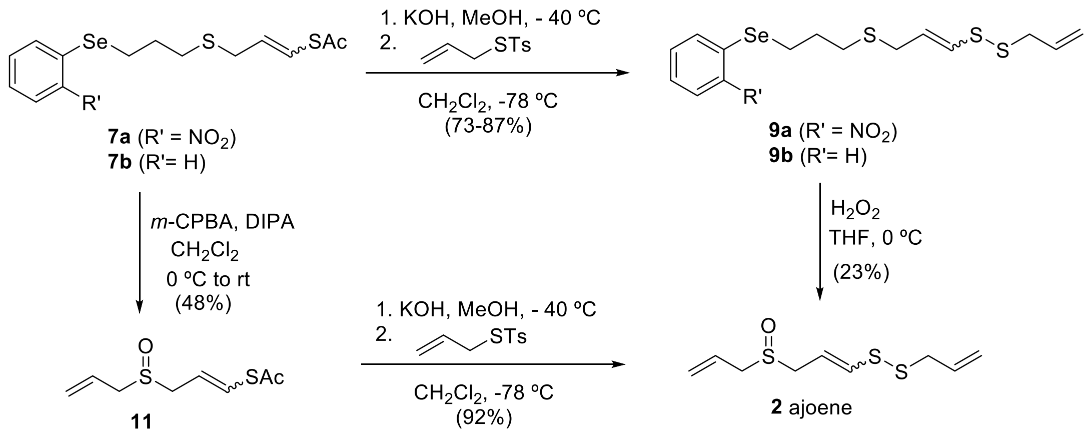

3. Experimental part

3-(Prop-2-yn-1-ylthio)propan-1-ol (4):

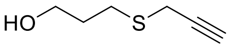

To a stirred solution of KOH (0.324 g, 5.60 mmol) in degassed CH_3OH (5.6 mL) at 0 °C, the isothiouronium salt **S1** (0.303 g, 2.24 mmol) was added. After 30 min, propargyl bromide (80% in toluene, 0.35 mL, 3.36 mmol) was added dropwise and the mixture left to warm up gradually to room temperature. The methanol was removed under vacuum, H_2O (5 mL) was added and the residue extracted with CH_2Cl_2 (2 x 10 mL). Following drying and removal of solvent, the residue was purified by column chromatography using Biotage Isolera (gradient: 100 % hexane for 3 column volume (CV), then increased to 90:10 hexane: ethyl acetate over 5 CV and held over 3 CV), giving the propargylic alcohol **4** (0.157 g, 56% yield) as a colourless oil.

^{1}H NMR (400 MHz, $CDCl_3$): δ = 1.65 (bs, 1H, O*H*), 1,78 − 2.03 (m, 2H, C*H*$_2$), 2.24 (t, *J* = 2.6 Hz, 1H, $CH_2C{\equiv}CH$), 2.68 − 2.91 (m, 2H, C*H*$_2$), 3.18-3-3.33 (m, 2H, C*H*$_2$C≡CH), 3.77 (td, *J* = 6.1, 1.4 Hz, 2H, C*H*$_2$) ppm. ^{13}C NMR (100 MHz, $CDCl_3$): δ = 19.3 (*C*H$_2$C≡CH), 28,4 (*C*H$_2$), 31.5 (*C*H$_2$), 71.2 (CH$_2$C≡*C*H); 80,0 (CH$_2$*C*≡CH) ppm. HRMS (NSI): $[M+H]^+$ calc. 164.0740, found 164.0739 $[C_6H_{11}OS{\bullet}NH_3OH]^+$. IR (neat): 3298, 1265, 1045, 733 cm^{-1}.

(3-Bromopropyl)(phenyl)selane (5):

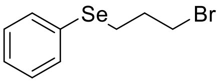

Procedure. To a stirred solution of diphenyl diselenide (0.312 g, 1 mmol) in ethanol (5 mL), sodium borohydride (0.076 g, 2 mmol) was added portionwise at 0 °C under nitrogen atmosphere. During this process, the yellow colour of the reaction mixture faded away. Then 1,3-dibromopropane (0.4 mL, 4 mmol) was added dropwise, and the reaction mixture was stirred for 2 h. After completion, water (10 mL) was added to the reaction and the resulting mixture extracted with diethyl ether (3 x 10 mL). The organic layer was washed with brine (20 mL), dried over Mg_2SO_4, and the solvent evaporated. The crude was purified by chromatography using Biotage Isolera (gradient: 100 % hexane for 10 column volume (CV), then increased to 90:10 hexane: ethyl acetate over 10 CV) to give compound **5** (0.512 g, 92% yield) as a colourless oil.

^{1}H NMR (400 MHz, $CDCl_3$): δ = 2.15 − 2.23 (m, 2H, C*H*$_2$), 3.03 (t, *J* = 6.5 Hz, 2H, C*H*$_2$), 3.51 (t, *J* = 6.5, 2H, C*H*$_2$), 7.24 − 7.31 (m, 3H, Ar*H*), 7.48 − 7.54 (m, 2H, Ar*H*) ppm. ^{13}C NMR (100 MHz, $CDCl_3$): δ = 25.9 (*C*H$_2$), 32.7 (*C*H$_2$), 33.2 (*C*H$_2$), 127.3 (C_{Ar}H), 129.3 (C_{Ar}H), 129.5 (C_{Ar}), 133.0 (C_{Ar}H) ppm. The spectroscopic data are in agreement with literature.[1]

General Procedure. Thiourea (0.198 g, 2.60 mmol) was dissolved in acetonitrile (5 mL) under N_2 atmosphere and the alkylbromide (2 mmol) was added to the solution which was then refluxed for 2 h. The reaction was cooled in an ice-bath to afford a white solid product that was filtered on a Büchner funnel. The product was

washed with ice-cold acetonitrile (20 mL) and dried further on the high vacuum pump to yield the isothiouronium salt as a colourless solid.

2-(3-Hydroxypropyl)isothiouronium bromide (S1):

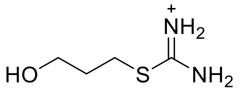

Synthesized according to the general procedure using 3-bromo-1-propanol (0.18 mL, 2 mmol). The isothiouronium salt **S1** (0.344 g, 80% yield) was obtained as a colourless solid.

^{1}H NMR (400 MHz, CH_3OD): δ = 1.93 (t, *J*= 6.5 Hz, 2H, C*H*$_2$), 3.26 (t, *J*= 6.5, 2H, C*H*$_2$), 3.69 (t, *J*= 6.5, 2H, C*H*$_2$), 4.86 - 4.83 (m, 5H, -N*H*$_2$ an -O*H*) ppm. ^{13}C NMR (100 MHz, CH_3OD): δ = 28.6 (*C*H$_2$), 32.4 (*C*H$_2$), 60.1 (*C*H$_2$), 173.3 (*C*(NH$_2$)$_2$) ppm. HRMS (ESI) [M-Br]$^+$ calc. 135.0587, found 135.0583 [$C_4H_{11}N_2OS$]$^+$. IR (neat): 3184, 3063, 1651, 1634, 1040, 1016, 914, 474 cm^{-1}. m.p. **=** 89 – 91 °C.

2-(3-(Phenylselanyl)propyl)isothiouronium bromide (S2):

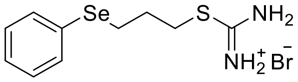

Synthesized according to the general procedure using **5** (0.512 g, 3.7 mmol). The isothiouronium salt **S2** (1.06 g, 81% yield) was obtained as a colourless solid.

^{1}H NMR (400 MHz, CH_3OD): δ = 2.01 (p, *J* = 7.2 Hz, 2H, C*H*$_2$), 3.01 (t, *J* = 7.2 Hz, 2H, C*H*$_2$), 3.23 (t, *J* = 7.2 Hz, 2H, C*H*$_2$), 7.24 − 7.29 (m, 3H, Ar*H*), 7.49 − 7.52 (m, 2H, Ar*H*) ppm. ^{13}C NMR (100 MHz, CH_3OD): δ = 26.1 (*C*H$_2$), 30.2 (*C*H$_2$), 31.5 (*C*H$_2$), 128.3 (C_{Ar}H), 130.3 (C_{Ar}H), 130.5 (C_{Ar}), 140.0 (C_{Ar}), 172.6 (*C*(NH$_2$)$_2$) ppm. HRMS (NSI): [M-Br]$^+$ calc. 275.0116, found 275.0112 [$C_{10}H_{15}N_2SSe$]$^+$. IR(neat): 3057, 1647, 1435, 1070, 737, 688, 460 cm^{-1}. m.p. = 126 – 128 °C.

(3-((2-Nitrophenyl)selanyl)propyl)(prop-2-yn-1-yl)sulfane (6a):

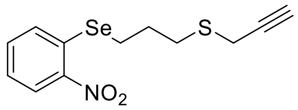

Alcohol **4** (0.195 g, 1.5 mmol) and 2-nitrophenylselenium cyanate (0.340 g, 1.5 mmol) were dissolved in THF (5 mL) under argon atmosphere at room temperature. Tributylphosphine (0.38 mL, 1.5 mmol) was added and the reaction mixture was left stirring for 2 h. After completion, the reaction was quenched with NH_4Cl solution (10 mL) and extracted with ethyl acetate (3 x 20 mL). The combined organic phases were dried over Mg_2SO_4, filtered and concentrated. The crude product was purified by column chromatography using Biotage Isolera

(gradient: 100% hexane for 3 column volume (CV), then increased to 90:20 hexane: ethyl acetate over 15 CV and held over 3 CV) to give compound **6a** (306 mg, 65% yield) as a yellow oil.

^{1}H NMR (400 MHz, $CDCl_3$): δ = 2.08 (p, *J* = 7.0 Hz, 2H, C*H*$_2$), 2.24 (t, *J* = 2.6 Hz, 1H, $CH_2C{\equiv}CH$), 2.85 (t, *J* = 7.0, 2H, C*H*$_2$), 3.03 (t, *J* = 7.0 Hz, 2H, C*H*$_2$), 3.25 (d, *J* = 2.6, 2H, $CH_2C{\equiv}CH$), 7.31 (ddd, *J* = 8.4, 6.0, 2.4 Hz, 1H, Ar*H*), 7.48 − 7.59 (m, 2H, Ar*H*), 8.24 − 8.29 (m, 1H, Ar*H*) ppm. ^{13}C NMR (100 MHz, $CDCl_3$): δ = 19.3 ($CH_2C{\equiv}CH$), 24.6 (CH_2), 27.5 (CH_2), 31.7 (CH_2), 71.4 ($CH_2C{\equiv}CH$), 79.7 ($CH_2C{\equiv}CH$), 125.5 ($C_{Ar}H$), 126.5 ($C_{Ar}H$), 129.0 ($C_{Ar}H$), 133.1 ($C_{Ar}Se$), 133.7 ($C_{Ar}H$), 146.9 ($C_{Ar}NO_2$) ppm. HRMS (ASAP): $[M+NH_4]^+$ calc. 333.0176, found 333.0172 $[C_{12}H_{17}N_2O_2SSe]^+$. IR (neat): 3298, 2939, 2862, 1512, 1330, 908, 729 cm^{-1}.

(3-(Phenylselanyl)propyl)(prop-2-yn-1-yl)sulfane (6b):

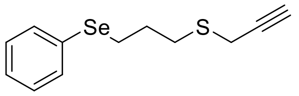

Synthesized according to the general procedure using isothiouronium salt **S2** (0.885 g, 2.5 mmol); **6b** (0.838 g, 84% yield) was obtained as a colourless solid.

^{1}H NMR (400 MHz, $CDCl_3$): δ = 2.00 (p, *J* = 7.1 Hz, 2H, C*H*$_2$), 2.22 (t, *J* = 2.6 Hz, 1H, $CH_2C{\equiv}CH$), 2.80 (t, *J* = 7.2, 2H, C*H*$_2$), 3.01 (t, *J* = 7.2 Hz, 2H, C*H*$_2$), 3.21 (d, *J* = 2.6, 2H, $CH_2C{\equiv}CH$), 7.23 − 7.29 (m, 3H, Ar*H*), 7.49 − 7.51 (m, 2H, Ar*H*) ppm. ^{13}C NMR (100 MHz, $CDCl_3$): δ = 19.3 ($CH_2C{\equiv}CH$), 26.5(CH_2), 29.2 (CH_2), 31.4 (CH_2), 71.2 ($CH_2C{\equiv}CH$), 80.0 ($CH_2C{\equiv}CH$), 127.1 ($C_{Ar}H$), 129.2($C_{Ar}H$), 130.0 ($C_{Ar}Se$), 132.9 ($C_{Ar}H$) ppm. HRMS (ASAP): $[M+H]^+$ calc. 271.0053, found 271.0059 $[C_{12}H_{15}SSe]^+$. IR (neat): 1477, 1238,1022, 906, 731 cm^{-1}.

(*E/Z*)-3-((3-((2-Nitrophenyl)selanyl)propyl)thio)prop-1-en-1-yl) ethanethioate (7a):

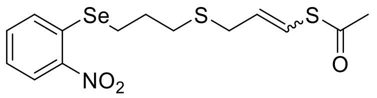

The propargylic sulfide **6a** (0.300 g, 0.96 mmol) was dissolved in degassed toluene (2 mL) and the solution heated to 85 °C under N_2. AIBN (15.8 mg, 10 mol%) or ACCN (23.4 mg, 10 mol%) was added to the solution directly, followed by the dropwise addition of thioacetic acid (75 µL, 1.06 mmol) in toluene (1 mL) over 40 minutes using a syringe pump. The mixture was left stirring for 1 h. The reaction was then quenched with aqueous saturated solution of sodium carbonate (3 mL) and the toluene removed under vacuum. The remaining residue was extracted with CH_2Cl_2 (2 x 10 mL) and the combined extracts were washed with brine (2 x 10 mL) and dried over $MgSO_4$. The solvent was removed under vacuum and the resulting residue purified by column chromatography using Biotage Isolera (gradient: 100% hexane for 3 column volume (CV), then increased to 80:20 hexane: diethyl ether over 15 CV, then hexane: diethyl ether over 3 CV) to afford compound **7a** (AIBN: 0.112 g, 30% yield; ACCN: 0.187 g, 50% yield, d.r. *E/Z* = 0.47:1) as a yellow oil.

E-isomer: ^{1}H NMR (400 MHz, $CDCl_3$): δ = 1.97-2.08 (m, 2H, C*H*$_2$), 2.35 (s, 3H, C*H*$_3$), 2.59-2.65 (m, 2H, C*H*$_2$), 3.02 (t, *J* = 8.0 Hz, 2H, C*H*$_2$), 3.22 (dd, *J* = 7.5, 1.2 Hz, 2H, $CH_2CH{=}CH$), 5.77-5.87 (m, 1H, $CH_2CH{=}CH$), 6.53

(dt, *J* = 15.6, 1.2 Hz, 1H, CH_2CH=C*H*), 7.29 − 7.34 (m, 1H, Ar*H*), 7.52 − 7.54 (m, 2H, Ar*H*), 8.27 − 8.29 (m, 1H, Ar*H*) ppm. ^{13}C NMR (100 MHz, $CDCl_3$): δ = 24.7 (*C*H_2), 28.0 (*C*H_2), 30.6 (*C*H_3), 30.9 (*C*H_2), 34.2 (*C*H_2CH=CH), 119.7 (CH_2*C*H=CH), 125.6 (C_{Ar}H), 126.7 (C_{Ar}H), 129.1 (C_{Ar}H), 129.9 (CH_2CH=*C*H), 133.4 (C_{Ar}Se), 133.8 (C_{Ar}H), 147.0 ($C_{Ar}NO_2$), 193.0 (*C*=O) ppm; HRMS (ESI): [M+Na]$^+$ calc. 413. 9712, found 413.9703 [$C_{14}H_{17}NO_3S_2SeNa$]$^+$. IR (neat): 1699, 1514, 1330, 1303, 904, 727 cm^{-1}.

Z-isomer: ^{1}H NMR (400 MHz, $CDCl_3$): δ = 1.97 − 2.08 (m, 2H, C*H*$_2$), 2.38 (s, 3H, C*H*$_3$), 2.59 − 2.65 (m, 2H, C*H*$_2$), 3.00 (t, *J* = 8.0 Hz, 2H, C*H*$_2$), 3.19 (dd, *J* = 7.7, 1.0 Hz, 2H, C*H*$_2$CH=CH), 5.77 − 5.87 (m, 1H, CH_2C*H*=CH), 6.67 (dt, *J* = 9.6, 0.9 Hz, 1H, CH_2CH=C*H*), 7.29 − 7.34 (m, 1H, Ar*H*), 7.52 − 7.54 (m, 2H, Ar*H*), 8.27 − 8.29 (m, 1H, Ar*H*) ppm. ^{13}C NMR (100 MHz, $CDCl_3$): δ = 24.8 (*C*H_2), 28.2 (*C*H_2), 31.0 (*C*H_2), 31.1 (*C*H_3), 31.2 (*C*H_2CH=CH), 119.9 (CH_2*C*H=CH), 125.6 (C_{Ar}H), 126.7 (C_{Ar}H), 128.4 (CH_2CH=*C*H), 129.1 (C_{Ar}H), 133.4 (C_{Ar}Se), 133.8 (C_{Ar}H), 147.0 ($C_{Ar}NO_2$), 191.3 (*C*=O) ppm. HRMS (ESI): [M+Na]$^+$ calc. 413. 9712, found 413.9703 [$C_{14}H_{17}NO_3S_2SeNa$]$^+$. IR (neat): 1699, 1514, 1330, 1303, 904, 727 cm^{-1}.

(*E/Z*)-3-((3-(Phenylselanyl)propyl)thio)prop-1-en-1-yl) ethanethioate (7b):

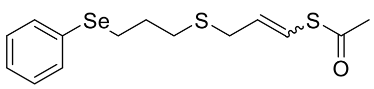

Synthesized according to the procedure shown for **7a** using propargylic sulfide **6b** (0.260 g, 0.96 mmol) as starting material. Product **7b** (AIBN: 0.235 g, 71% yield; ACCN: 0.211 g, 64%, d.r. *E/Z* = 0.50:1) was obtained as a pale-yellow oil.

E-isomer: ^{1}H NMR (400 MHz, $CDCl_3$): δ = 1.93 (quin, *J* = 8.0 Hz, 2H, C*H*$_2$), 2.36 (s, 3H, C*H*$_3$), 2.53 (t, *J* = 7.1, 2H, C*H*$_2$), 2.95 (td, *J* = 7.3, 3.6 Hz, 2H, C*H*$_2$), 3.14 (t, *J* = 8.3 Hz, 2H, C*H*$_2$CH=CH), 5.72 – 5.85 (m, 1H, CH_2C*H*=CH), 6.49 (d, *J* = 15.6 Hz, CH_2CH=C*H*, 1H), 7.24 – 7.26 (m, 3H, Ar*H*), 7.48 – 7.50 (m, 2H, Ar*H*) ppm. ^{13}C NMR (100 MHz, $CDCl_3$): δ = 26.6 (*C*H_2), 29.5 (*C*H_2), 30.5 (*C*H_2), 30.7 (*C*H_3), 34.1 (*C*H_2), 119.3 (CH_2CH=*C*H), 127.0 (C_{Ar}H), 129.1 (2C_{Ar}H), 130.1 (C_{Ar}Se), 130.2 (CH_2*C*H=CH), 132.8 (2C_{Ar}H), 192.9 (C=O) ppm. HRMS (ASAP): [M+Na]$^+$ calc. 347.0042, found 347.0042 [$C_{14}H_{19}OS_2Se$]$^+$. IR (neat): 1699, 1126, 736, 626 cm^{-1}.

Z-isomer: ^{1}H NMR (400 MHz, $CDCl_3$): δ = 1.93 (quin, *J* = 8.0 Hz, 2H, C*H*$_2$), 2.36 (s, 3H, C*H*$_3$), 2.53 (t, *J* = 7.1, 2H, C*H*$_2$), 2.95 (td, *J* = 7.3, 3.6 Hz, 2H, C*H*$_2$), 3.14 (t, *J* = 8.3 Hz, 2H, C*H*$_2$CH=CH), 5.72 – 5.85 (m, 1H, CH_2C*H*=CH), 6.65 (d, *J* = 9.6 Hz, 1H, CH_2CH=C*H*), 7.24 – 7.26 (m, 3H, Ar*H*), 7.48 – 7.50 (m, 2H, Ar*H*) ppm. ^{13}C NMR (100 MHz, $CDCl_3$): δ = 26.5 (*C*H_2), 29.8 (*C*H_2), 30.9 (*C*H_2), 31.0 (*C*H_3), 119.6 (CH_2CH=*C*H), 127.0 (C_{Ar}H), 128.6 (CH_2*C*H=CH), 129.1 (2C_{Ar}H), 130.1 (C_{Ar}Se), 132.8 (2C_{Ar}H), 191.3 (*C*=O) ppm. HRMS (ASAP): [M+Na]$^+$ calc. 347.0042, found 347.0042 [$C_{14}H_{19}OS_2Se$]$^+$. IR (neat): 1699, 1126, 736, 626 cm^{-1}.

(*E/Z*)-1-Allyl-2-(3-((3-((2-nitrophenyl)selanyl)propyl)thio)prop-1-en-1-yl)disulfane (9a):

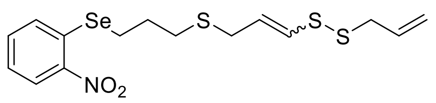

The vinyl thioacetate **7a** (0.190 g, 0.46 mmol) was dissolved in degassed CH_3OH (0.5 mL) and the solution was cooled to –40 ºC and stirred under N_2. KOH (26.9 mg, 0.48 mmol) in degassed methanol (0.5 mL) was added slowly and the reaction left stirring for 45 minutes, after which it was cooled down to –78 ºC. S-allyl 4-methylbenzenesulfonothioate (0.116 g, 0.51 mmol) dissolved in CH_2Cl_2 (0.5 mL) was added and the reaction allowed to stir for an hour at –78 ºC before being allowed to warm up. The reaction was quenched with saturated ammonium chloride (5 mL), the solvents were removed under vacuum on the rotary evaporator, and the resulting residue extracted with CH_2Cl_2 (3 x 10 mL). The combined extracts were washed with brine (2 x 10 mL) and dried over $MgSO_4$. The solvent was removed under vacuum and the resulting residue purified by column chromatography using Biotage Isolera (gradient: 100% hexane for 3 column volume (CV), then increased to 90:10 hexane: diethyl ether over 20 CV, then 90:10 hexane: diethyl ether over 3 CV) to give compound **9a** (0.141 g, 73% yield, d.r. *E/Z* = 0.50:1) as a yellow oil.

E-isomer: ^{1}H NMR (400 MHz, $CDCl_3$): δ = 1.99 − 2.12 (m, 2H, C*H*$_2$), 2.60 − 2.66 (m, 2H, C*H*$_2$), 3.03 (t, *J* = 7.5 Hz, 2H, C*H*$_2$), 3.21 (d, *J* = 7.8 Hz, 2H, C*H*$_2$CH=CH), 3.34 (d, *J* = 7.5 Hz, 2H, C*H*$_2$CH=CH$_2$), 5.12 − 5.23 (m, 2H, CH$_2$CH=C*H*$_2$), 5.78 − 5.89 (m, 2H, CH$_2$C*H*=CH2 and CH$_2$C*H*=CH), 6.11 (d, *J* = 14.7 Hz, 1H, CH$_2$CH=C*H*), 7.30 − 7.35 (m, 1H, Ar*H*), 7.51 − 7.56 (m, 2H, Ar*H*), 8.29 (dd, *J* = 8.5, 1.5 Hz, 1H, Ar*H*) ppm. ^{13}C NMR (100 MHz, $CDCl_3$): δ = 24.6 (*C*H$_2$), 27.9 (*C*H$_2$), 30.9 (*C*H$_2$), 33.6 (*C*H$_2$CH=CH), 41.3 (*C*H$_2$CH=CH$_2$), 119.0 (CH$_2$CH=*C*H$_2$), 125.5 (*C*$_{Ar}$H), 126.6 (*C*$_{Ar}$H), 127.6 (CH$_2$CH=*C*H), 128.2 (CH$_2$CH=CH), 129.0 (C$_{Ar}$H), 132.7 (CH$_2$*C*H=CH$_2$), 133.1 (*C*$_{Ar}$Se), 133.6 (*C*$_{Ar}$H), 147.0 (C$_{Ar}$NO$_2$) ppm. HRMS (ASAP): [M+H]$^+$ calc. 421. 9820, found 421.9823 [$C_{15}H_{20}NO_2S_3Se$]$^+$. IR (neat): 1589, 1566, 1514, 1330, 1303, 731 cm^{-1}.

Z-isomer: ^{1}H NMR (400 MHz, $CDCl_3$): δ = 1.99 − 2.12 (m, 2H, C*H*$_2$), 2.60 − 2.66 (m, 2H, C*H*$_2$), 3.03 (t, *J* = 7.5 Hz, 2H, C*H*$_2$), 3.26 (d, *J* = 7.8 Hz, 2H, C*H*$_2$CH=CH), 3.36 (d, *J* = 7.5 Hz, 2H, C*H*$_2$CH=CH$_2$), 5.12 − 5.23 (m, 2H, CH$_2$CH=C*H*$_2$), 5.62 − 5.72 (m, 1H, CH$_2$C*H*=CH), 5.78 − 5.89 (m, 1H, CH$_2$C*H*=CH$_2$), 6.25 (d, *J* = 9.3, 1H, CH$_2$CH=C*H*), 7.30 − 7.35 (m, 1H, Ar*H*), 7.51 − 7.56 (m, 2H, Ar*H*), 8.29 (dd, *J* = 8.5, 1.5 Hz, 1H, Ar*H*) ppm. ^{13}C NMR (100 MHz, $CDCl_3$): δ = 24.7 (*C*H$_2$), 28.3 (*C*H$_2$), 29.4 (*C*H$_2$CH=CH), 31.2 (*C*H$_2$), 45.1 (*C*H$_2$CH=CH$_2$), 119.7 (CH$_2$CH=*C*H$_2$), 125.4 (*C*$_{Ar}$H), 126.5 (*C*$_{Ar}$H), 127.9 (CH$_2$CH=*C*H), 129.0 (*C*$_{Ar}$H), 132.4 (CH$_2$CH=*C*H), 132.8 (CH$_2$*C*H=CH$_2$), 133.2 (*C*$_{Ar}$Se), 133.6 (*C*$_{Ar}$H), 147.0 (*C*$_{Ar}$NO$_2$) ppm. HRMS (ASAP): [M+H]$^+$ calc. 421.9820, found 421.9823 [$C_{15}H_{20}NO_2S_3Se$]$^+$. IR (neat): 1589, 1566, 1514, 1330, 1303, 731 cm^{-1}.

(*E/Z*)-1-Allyl-2-(3-((3-(phenylselanyl)propyl)thio)prop-1-en-1-yl)disulfane (9b):

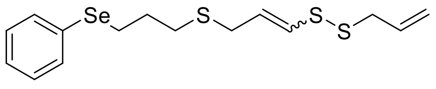

Synthesized according to the procedure shown for **9a** using **7b** (0.160 g, 0.46 mmol) as starting material. Product **9b** (0.150 g, 87% yield, d.r. *E/Z* = 0.64:1) was obtained as a pale-yellow oil.

E-isomer: ^{1}H NMR (400 MHz, $CDCl_3$): δ = 1.91 − 2.03 (m, 2H, C*H*$_2$), 2.56 − 2.63 (m, 2H, C*H*$_2$), 3.00 (t, *J* = 7.3 Hz, 2H, C*H*$_2$), 3.16 (d, *J* = 7.4 Hz, 2H, C*H*$_2$CH=CH), 3.35 (d, *J* = 7.1 Hz, 2H, C*H*$_2$CH=CH$_2$), 5.13 − 5.23 (m, 2H, CH$_2$CH=C*H*$_2$), 5.80 − 5.90 (m, 2H, CH$_2$C*H*=CH$_2$ and CH$_2$C*H*=CH), 6.08 (d, *J* = 14.7, 1H, CH$_2$CH=C*H*), 7.25 − 7.31 (m, 3H, Ar*H*), 7.50 − 7.52 (m, 2H, Ar*H*) ppm. ^{13}C NMR (100 MHz, $CDCl_3$): δ = 26.5 (*C*H$_2$), 29.6 (*C*H$_2$), 30.7 (*C*H$_2$), 33.5 (*C*H$_2$), 41.4 (*C*H$_2$), 119.1 (CH$_2$CH=*C*H$_2$), 127.1 (*C*$_{Ar}$H), 127.9 (CH$_2$CH=*C*H), 128.0 (CH$_2$*C*H=*C*H), 129.2 (2*C*$_{Ar}$H), 130.0 (*C*$_{Ar}$Se), 132.8 (2*C*$_{Ar}$H), 132.9 (CH$_2$*C*H=CH$_2$) ppm. HRMS (ASAP): $[M+H]^+$ calc. 376.9969, found 376.9969 $[C_{15}H_{21}S_3Se]^+$. IR (neat): 1577, 1477, 1072, 1022, 910, 732, 690 cm^{-1}.

Z-isomer: ^{1}H NMR (400 MHz, $CDCl_3$): δ = 1.91 − 2.03 (m, 2H, C*H*$_2$), 2.56 − 2.63 (m, 2H, C*H*$_2$), 3.00 (t, *J* = 7.3 Hz, 2H, C*H*$_2$), 3.25 (d, *J* = 7.8 Hz, 2H, C***H*$_2$**CH=CH), 3.33 (d, *J* = 7.1 Hz, 2H, C*H*$_2$CH=CH$_2$), 5.13 − 5.23 (m, 2H, CH$_2$CH=C*H*$_2$), 5.60 − 5.71 (m, 1H, CH$_2$C*H*=CH), 5.80 − 5.90 (m, 1H, CH$_2$C*H*=CH$_2$), 6.23 (d, *J* = 9.3, 1H, CH$_2$CH=C*H*), 7.25 − 7.31 (m, 3H, Ar*H*), 7.50 − 7.52 (m, 2H, Ar*H*) ppm. ^{13}C NMR (100 MHz, $CDCl_3$): δ = 26.6 (*C*H$_2$), 29.3 (*C*H$_2$CH=CH), 29.9 (*C*H$_2$), 31.1 (*C*H$_2$), 42.2 (*C*H$_2$CH=CH$_2$), 119.2 (CH$_2$CH=*C*H$_2$), 127.0 (*C*$_{Ar}$H), 128.3 (CH$_2$CH=CH), 129.3 (2*C*$_{Ar}$H), 130.1 (*C*$_{Ar}$Se), 132.3 (CH$_2$CH=*C*H), 132.8 (2*C*$_{Ar}$H), 132.9 (CH$_2$*C*H=CH$_2$) ppm. HRMS (ASAP): $[M+H]^+$ calc. 376.9969, found 376.9969 $[C_{15}H_{21}S_3Se]^+$. IR (neat): 1577, 1477, 1072, 1022, 910, 732, 690 cm^{-1}.

(*E/Z*)-1-Allyl-2-(3-(allylsulfinyl)prop-1-en-1-yl)disulfane (Ajoene) (2):

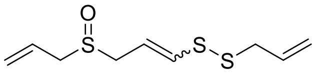

General procedure: The vinyl disulfide **9a** (0.140 g, 0.33 mmol) dissolved in THF (3 mL) was cooled to 0 °C under N_2, and H_2O_2 (30% w/w in H_2O, 0.075 mL, 0.66 mmol) added dropwise. The reaction was allowed to proceed for 1 h at 0 °C and then warmed to rt (2 h). Saturated $NaHCO_3$ aq. solution (5 mL) was added and the residue was extracted with EtOAc (2×10 mL). The combined fractions were washed with brine (2×10 mL) and dried over $MgSO_4$. The solvent was removed under vacuum and the resulting residue purified by column chromatography using Biotage Isolera (gradient 100% hexane for 2 column volume (CV), then increased to 20:80 hexane: diethyl ether over 10 CV, held over 5 CV, increased to 0:100 over 5 CV and held over 10 CV diethyl ether) to afford ajoene **2** (21 mg, 27%, d.r. *E/Z* = 0.56:1) as a pale yellow oil.

Following the general procedure, compound **7b** (0.120 g, 0.33 mmol) was also used to synthesise ajoene **2** (17.7 mg, 23%, d.r. *E/Z* = 0.60:1).

E-isomer: ^{1}H NMR (400 MHz, $CDCl_3$): δ = 3.29 (d, *J* = 7.6 Hz, 2H, C*H*$_2$CH=CH$_2$), 3.26 – 3.54 (m, 4H, C*H*$_2$CH=CH$_2$, C*H*$_2$CH=CH), 5.11 – 5.19 (m, 2H, CH$_2$CH=C*H*$_2$), 5.27 – 5.44 (m, 2H, CH$_2$CH=CH$_2$), 5.69 – 5.93 (m, 3H, CH$_2$C*H*=CH$_2$, CH$_2$C*H*=CH and CH$_2$C*H*=CH$_2$), 6.31 (d, *J* = 14.8 Hz, 1H, CH$_2$CH=C*H*) ppm. ^{13}C NMR (100 MHz, $CDCl_3$): δ = 41.3 (*C*H$_2$), 52.9 (*C*H$_2$), 54.4 (*C*H$_2$), 116.8 (CH$_2$*C*H=CH), 119.3 (CH$_2$CH=*C*H$_2$), 123.7 (CH$_2$CH=*C*H$_2$), 125.6 (CH$_2$*C*H=CH$_2$), 132.4 (CH$_2$CH=CH$_2$), 134.7 (CH$_2$CH=*C*H) ppm. The spectroscopic data are in agreement with literature.[2]

Z-isomer: ^{1}H NMR (400 MHz, $CDCl_3$): δ = 3.37 (d, *J* = 7.3 Hz, 2H, C*H*$_2$CH=CH$_2$), 3.39 – 3.67 (m, 4H, C*H*$_2$CH=CH$_2$, C*H*$_2$CH=CH), 5.11 – 5.24 (m, 2H, CH$_2$CH=C*H*$_2$), 5.35 – 5.50 (m, 2H, CH$_2$CH=C*H*$_2$), 5.69 – 5.99 (m, 3H, CH$_2$C*H*=CH$_2$, CH$_2$C*H*=CH and CH$_2$C*H*=CH$_2$), 6.56 (d, *J* = 9.5 Hz, 1H, CH$_2$CH=C*H*) ppm. ^{13}C NMR (100 MHz, $CDCl_3$): δ = 42.1 (*C*H$_2$), 49.6 (*C*H$_2$), 54.9 (*C*H$_2$), 118.0 (CH$_2$*C*H=CH), 119.3 (CH$_2$CH=*C*H$_2$), 123.7 (CH$_2$CH=*C*H$_2$), 125.6 (CH$_2$*C*H=CH$_2$), 132.6(CH$_2$*C*H=CH$_2$), 138.6 (CH$_2$CH=*C*H) ppm. IR (neat): 1037, 931, 733 cm^{-1}. The spectroscopic data are in agreement with literature.[2]

(*E/Z*)-S-(3-(Allylsulfinyl)prop-1-en-1-yl) ethanethioate (10):

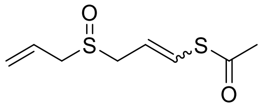

The vinyl thioacetate **7b** (0.200 g, 0.58 mmol) dissolved in CH_2Cl_2 (6 mL) was cooled to 0 °C under N_2, and *m*-CPBA (0.200 g, 1.16 mmol) was added in one portion. The reaction proceeded for 1 h at 0 °C and before being allowed to warm to room temperature, DIPA (0.16 mL, 1.16 mmol) was added. After 2 hours reaction, saturated $NaHCO_3$ aqueous solution (5 mL) was added and the residue was extracted with CH_2Cl_2 (3×10 mL). The combined fractions were washed with brine (2×10 mL) and dried over Mg_2SO_4. The solvent was removed under vacuum and the resulting residue purified by column chromatography using Biotage Isolera (gradient: 100% hexane for 2 column volume (CV), then increased to 20:80 hexane: ethyl acetate over 10 CV, held over 5 CV, increased to 0:100 over 5 CV and held over 10 CV ethyl acetate) to give compound **10** (57 mg, 48% yield, d.r. *E/Z* = 0.67:1) as a pale yellow oil.

E-isomer: ^{1}H NMR (400 MHz, $CDCl_3$): δ = 2.37 (s, 3H, C*H*$_3$), 3.32 – 3.68 (m, 4H, C*H*$_2$), 5.36 – 5.50 (m, 2H, CH$_2$CH=C*H*$_2$), 5.80 – 6.00 (m, 2H, CH$_2$C*H*=CH$_2$ and CH$_2$C*H*=CH), 6.85 (dt, *J* = 15.9, 1.2 Hz, 1H, CH$_2$CH=C*H*) ppm. ^{13}C NMR (100 MHz, $CDCl_3$): δ = 30.6 (CH$_3$), 53.5 (CH$_2$), 54.5 (CH$_2$), 120.3 (CH$_2$*C*H=CH$_2$), 124.1 (CH$_2$CH=*C*H$_2$), 125.5 (CH$_2$*C*H=CH), 126.2 (CH$_2$CH=*C*H), 192.1 (*C*=O) ppm. HRMS (NSI): [M+H]$^+$ calc. 205.0351, found 205.0352 [$C_8H_{13}O_2S_2$]$^+$. IR (neat): 1697, 1034, 952, 731, 621 cm^{-1}.

Z-isomer: ^{1}H NMR (400 MHz, $CDCl_3$): δ = 2.41 (s, 3H, C*H*$_3$), 3.32 – 3.68 (m, 4H, C*H*$_2$), 5.36 – 5.50 (m, 2H, CH$_2$CH=C*H*$_2$), 5.80 – 6.00 (m, 2H, CH$_2$C*H*=CH$_2$ and CH$_2$C*H*=CH), 7.01 (dt, *J* = 9.8, 1.0 Hz, 1H, CH$_2$CH=C*H*). ^{13}C NMR (100 MHz, $CDCl_3$): δ = 31.9 (*C*H$_3$), 51.3 (*C*H$_2$), 54.9 (*C*H$_2$), 118.6 (CH$_2$*C*H=CH$_2$), 124.0 (CH$_2$CH=*C*H$_2$), 125.6 (CH$_2$*C*H=CH$_2$), 126.1 (CH$_2$CH=*C*H), 190.5 (*C*=O) ppm. HRMS (NSI): [M+H]$^+$ calc. 205.0351, found 205.0352 [$C_8H_{13}O_2S_2$]$^+$. IR (neat): 1697, 1034, 952, 731, 621 cm^{-1}.

(*E/Z*)-S-(3-((3-(Phenylselanyl)propyl)sulfinyl)prop-1-en-1-yl) ethanethioate (11):

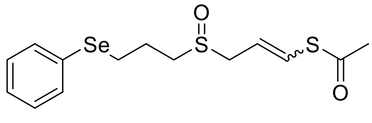

The vinyl thioacetate **7b** (0.100 g, 0.28 mmol) dissolved in mixture of CH_3OH (1.5 mL) and H_2O (0.45 mL) was cooled to 0 °C under N_2, and $NaIO_4$ (0.123 g, 0.57 mmol) was added in one portion. The reaction proceeded for 1 h at 0 °C and before being allowed to warm to room temperature. After 4 hours reaction, saturated $NaHCO_3$ aqueous solution (5 mL) was added and the residue was extracted with CH_2Cl_2 (3 × 10 mL). The combined fractions were washed with brine (2 × 5 mL) and dried over $MgSO_4$. The solvent was removed under vacuum and the resulting residue purified by column chromatography using Biotage Isolera (gradient: 100% hexane for 3 column volume (CV), increased to 10% ethyl acetate over 3 CV, then increased to 0:100 over 5 CV and held over 5 CV ethyl acetate) to give compound **11** (0.050 g, 50% yield, d.r. *E/Z* = 0.55:1) as a pale yellow oil.

E-isomer: ^{1}H NMR (400 MHz, $CDCl_3$): δ = 2.08 – 2.23 (m, 2H, C*H*$_2$), 2.38 (s, 3H, C*H*$_3$), 2.73 – 2.85 (m, 2H, C*H*$_2$), 3.02 (t, *J* = 7.0 Hz, 2H, C*H*$_2$), 3.36 – 3.66 (m, 2H, C*H*$_2$), 5.79 – 5.97 (m, 1H, CH_2C*H*=CH), 6.83 (dt, *J* = 15.9, 1.2 Hz, 1H, CH_2CH=C*H*), 7.20 – 7.32 (m, 3H, Ar*H*), 7.48 – 7.51 (m, 2H, Ar*H*) ppm. ^{13}C NMR (100 MHz, $CDCl_3$): δ = 23.3 (*C*H_2), 26.5 (*C*H_2), 30.6 (*C*H_3), 50.7 (*C*H_2), 55.3 (*C*H_2), 120.2 (CH_2*C*H=CH), 126.1 (CH_2CH=*C*H), 127.5 (C_{Ar}H), 129.3 (C_{Ar}Se), 129.4 (2C_{Ar}H), 133.2 (2C_{Ar}H), 192.0 (C=O) ppm. HRMS (ASAP): $[M+H]^+$ calc. 362.9991, found 362.9989 $[C_{14}H_{19}OS_2Se]^+$. IR (neat): 1699, 1126, 1045, 956, 736, 626 cm^{-1}.

Z-isomer: ^{1}H NMR (400 MHz, $CDCl_3$): δ = 2.08 – 2.23 (m, 2H, C*H*$_2$), 2.38 (s, 3H, C*H*$_3$), 2.73 – 2.85 (m, 2H, C*H*$_2$), 3.02 (t, *J* = 7.0 Hz, 2H, C*H*$_2$), 3.36 – 3.66 (m, 2H, C*H*$_2$), 5.79 – 5.97 (m, 1H, CH_2C*H*=CH), 7.01 (dt, *J* = 9.8, 1.0 Hz, 1H, CH_2CH=C*H*), 7.20 – 7.32 (m, 3H, Ar*H*), 7.48 – 7.51 (m, 2H, Ar*H*) ppm. ^{13}C NMR (100 MHz, $CDCl_3$): δ = 23.4 (*C*H_2), 26.6 (*C*H_2), 31.1 (*C*H_3), 50.9 (*C*H_2), 52.8 (*C*H_2), 118.6 (CH_2*C*H=CH), 126.2 (CH_2CH=*C*H), 127.4 (C_{Ar}H), 129.3 (C_{Ar}Se), 129.4 (2C_{Ar}H), 133.2 (2C_{Ar}H), 190.4 (C=O) ppm. HRMS (ASAP): $[M+H]^+$ calc. 362.9991, found 362.9989 $[C_{14}H_{19}OS_2Se]^+$. IR (neat): 1699, 1126, 1045, 956, 736, 626 cm^1.

_S_-Allyl 4-methylbenzenesulfonothioate (8):

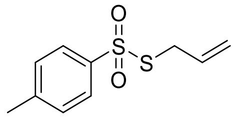

General procedure. Allyl chloride (0.16 mL, 2 mmol) and potassium *p*-toluenethiosulfonate (0.588 g, 2.6 mmol) were dissolved in DMF (3 mL) and stirred at room temperature for 2 h. The solution was then suspended in water (20 mL), which was extracted with EtOAc (2×10 mL). The combined extracts were washed with water (2×20 mL) to remove any residual DMF. Following drying over $MgSO_4$ and solvent evaporation the product was obtained as a colourless oil (0.319 g, 70% yield).

^{1}H NMR (400 MHz, $CDCl_3$): δ = 2.45 (s, 3H, C*H*$_3$), 3.67 (dt, *J* = 7.1, 1.2 Hz, 2H, C*H*$_2$C=C*H*$_2$), 5.11 (dd, *J* = 10.0, 0.9 Hz, 1H, CH_2C*H*=C*H*$_2$), 5.20 (dd, *J* = 16.9, 1.2 Hz, 1H, CH_2CH=C*H*$_2$), 5.62 – 5.79 (m, 1H, CH_2C*H*=CH_2), 7.34 (d, *J* = 8.0 Hz, 2H, Ar*H*), 7.81 (d, *J* = 8.0 Hz, 2H, Ar*H*) ppm. ^{13}C NMR (100 MHz, $CDCl_3$): δ = 21.8 (*C*H_3), 38.9 (*C*H_2CH=CH_2), 120.1 (CH_2CH=*C*H_2), 127.3 (2C_{Ar}H), 129.9 (2C_{Ar}H), 130.7 (CH_2*C*H=CH_2), 142.2 (C_{Ar}), 144.9 (C_{Ar}) ppm. The spectroscopic data is in agreement with literature.[3]

4. Gene reporter assays to determine QSI effect

An overnight culture of the *lasB-gfp* reporter strain was diluted into ABT minimal media with selective antibiotics to an OD600 of 0.08 and added to ajoene dilutions in a black, clear bottom microplate (Corning, CoStar). No compound was added to the first column of the plate and was used as a reference. Fluorescence Intensity (FI; excitation 485 nm, emission 540 nm) and cell density (OD 600nm) were read every 15 min for 16 hours (BMG Fluostar Omega), with a constant temperature of 37 ºC. The FI was normalised to cell density (FI/OD) before max FI output was compared between the reference and treated cultures and an inhibition value was calculated. This inhibition value was used to calculate an IC50 response using Prism 7 software (Graphpad).

5. References

[1] C. Schnabel, M. Hiersemann, *Org. Lett.* **2009**, *11*, 2555–2558.

[2] E. Block, S. Ahmad, M. K. Jain, R. W. Crecely, R. Apitz-Castro, M. R. Cruz, *J. Am. Chem. Soc.* **1984**, *106*, 8295–8296.

[3] C. H. Kaschula, R. Hunter, N. Stellenboom, M. R. Caira, S. Winks, T. Ogunleye, P. Richards, J. Cotton, K. Zilbeyaz, Y. Wang, V. Siyo, E. Ngarande, M. I. Parker, *Eur. J. Med. Chem.* **2012**, *50*, 236–254.

[4] a) T. H. Jakobsen, M. van Gennip, R. K. Phipps, M. S. Shanmugham, L. D. Christensen, M. Alhede, M. E. Skindersoe, T. B. Rasmussen, K. Friedrich, F. Uthe, P. Ø. Jensen, C. Moser, K. F. Nielsen, L. Eberl, T. O. Larsen, D. Tanner, N. Høiby, T. Bjarnsholt, M. Givskova, *Antimicrob. Agents Chemot.* **2012**, *56*, 2314–2325; b) T. H. Jakobsen, A. N. Warming, R. M. Vejborg, J. A. Moscoso, M. Stegger, F. Lorenzen, M. Rybtke, J. B. Andersen, R. Petersen, P. S. Andersen, T. E. Nielsen, T. Tolker-Nielsen, A. Filloux, H. Ingmer, M. Givskov, *Sci. Rep.* **2017**, *7*, 9857.

6. NMR spectra

(3-Bromopropyl)(phenyl)selane (5):

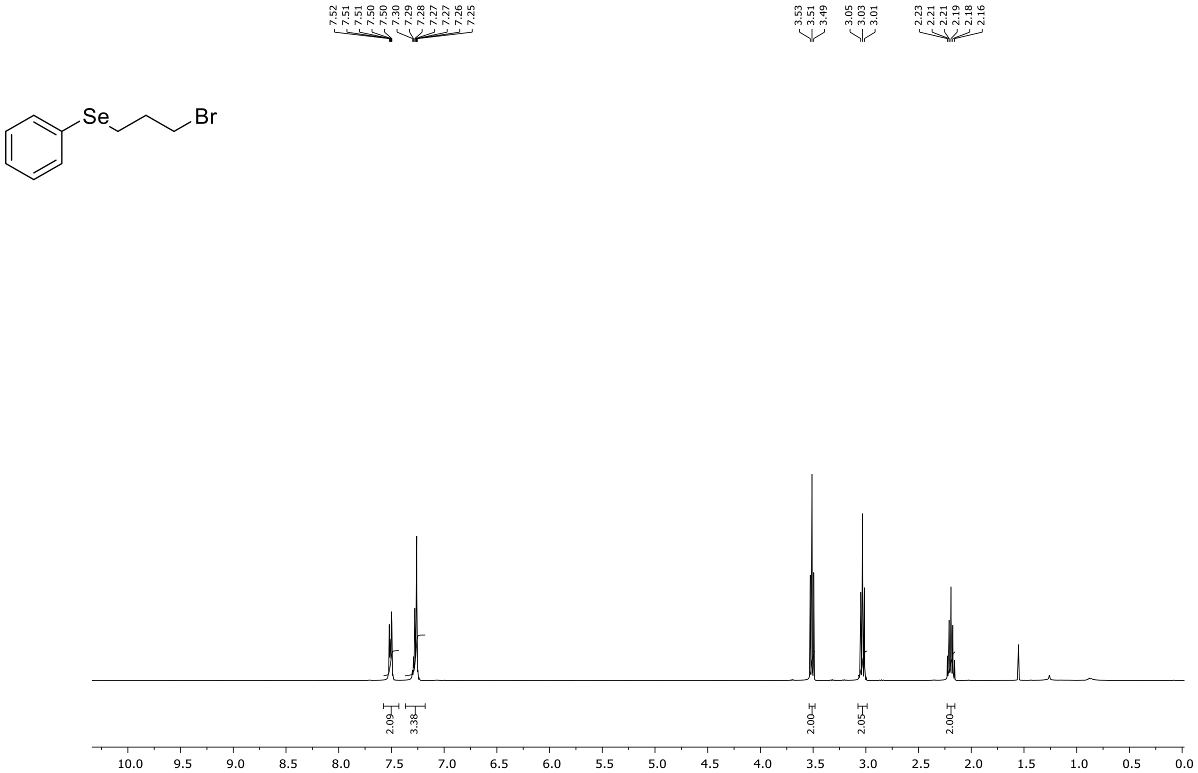

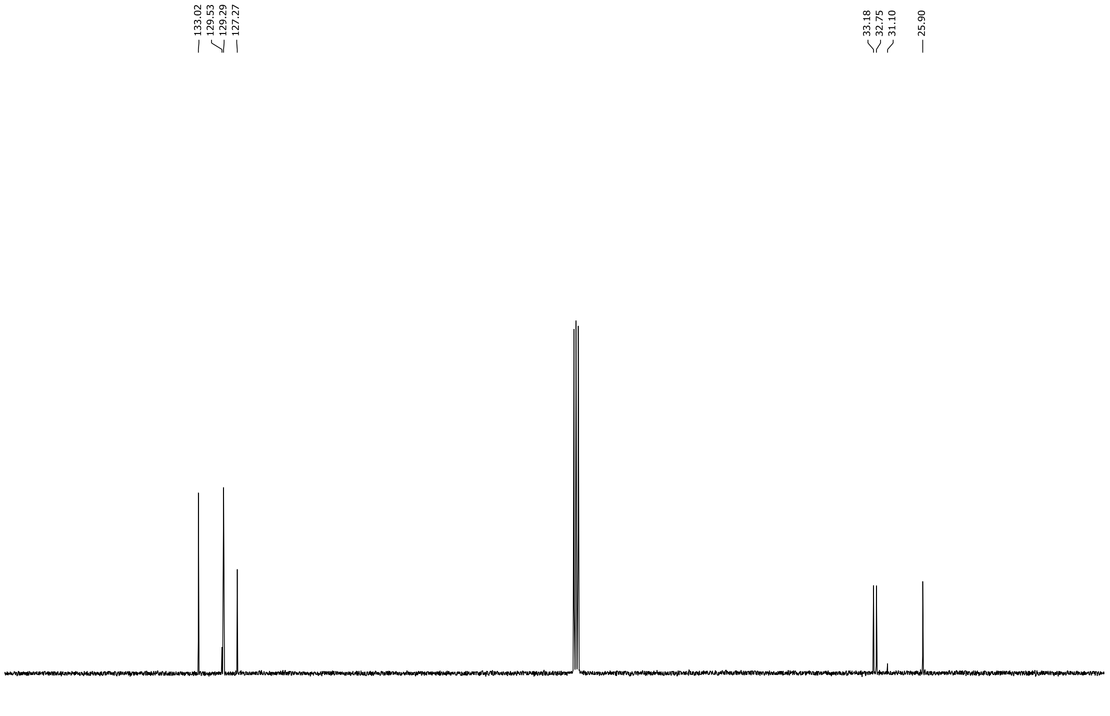

2-(3-(Phenylselanyl)propyl)isothiouronium bromide (S2):

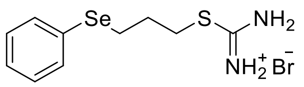

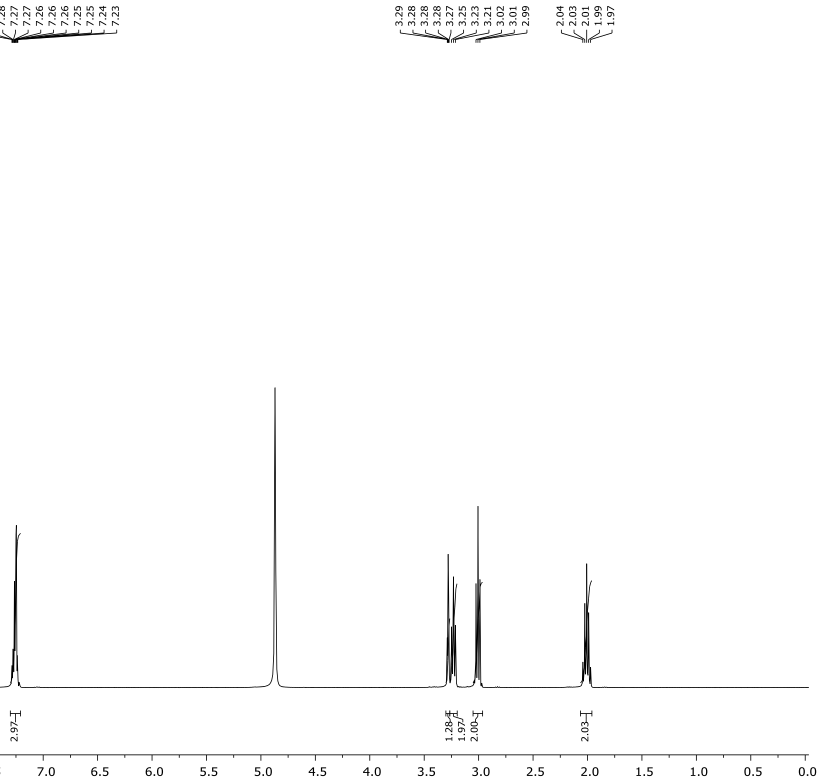

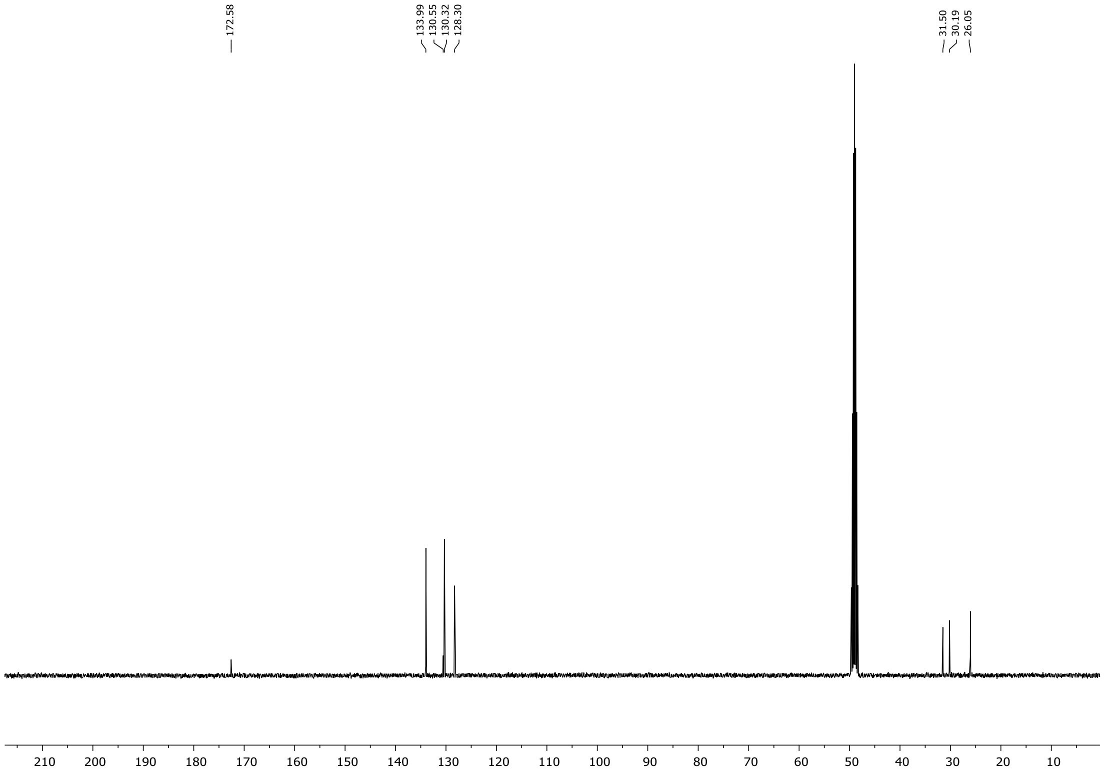

3-(Prop-2-yn-1-ylthio)propan-1-ol (4):

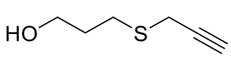

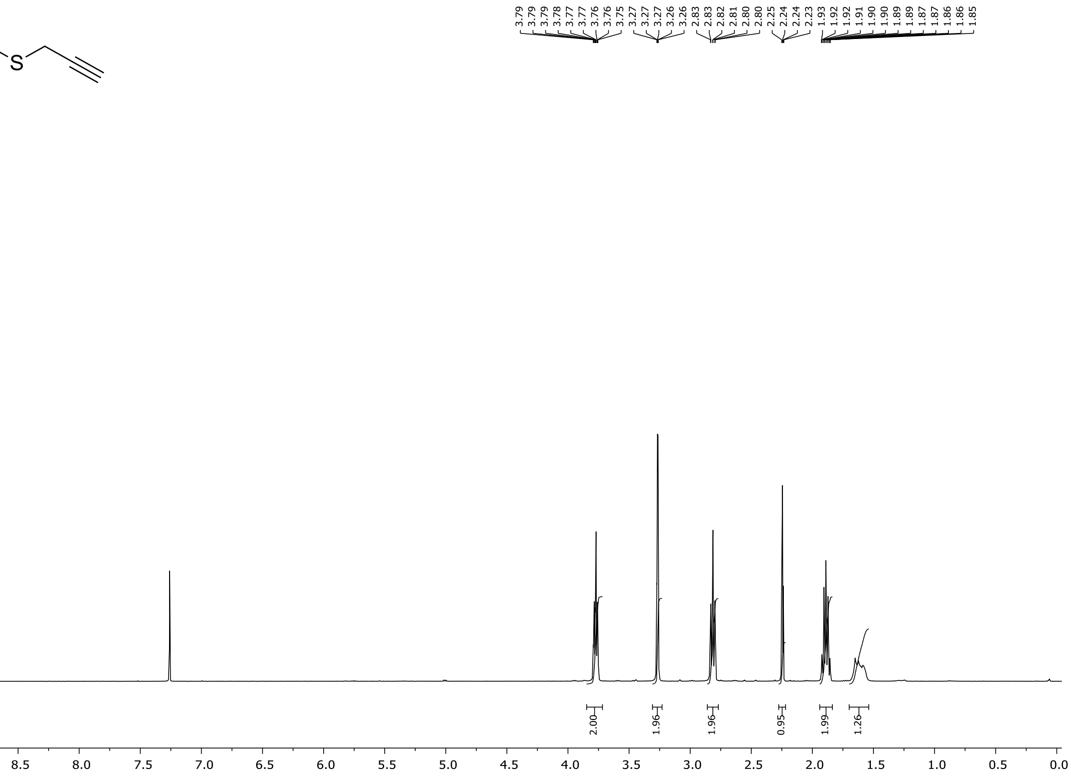

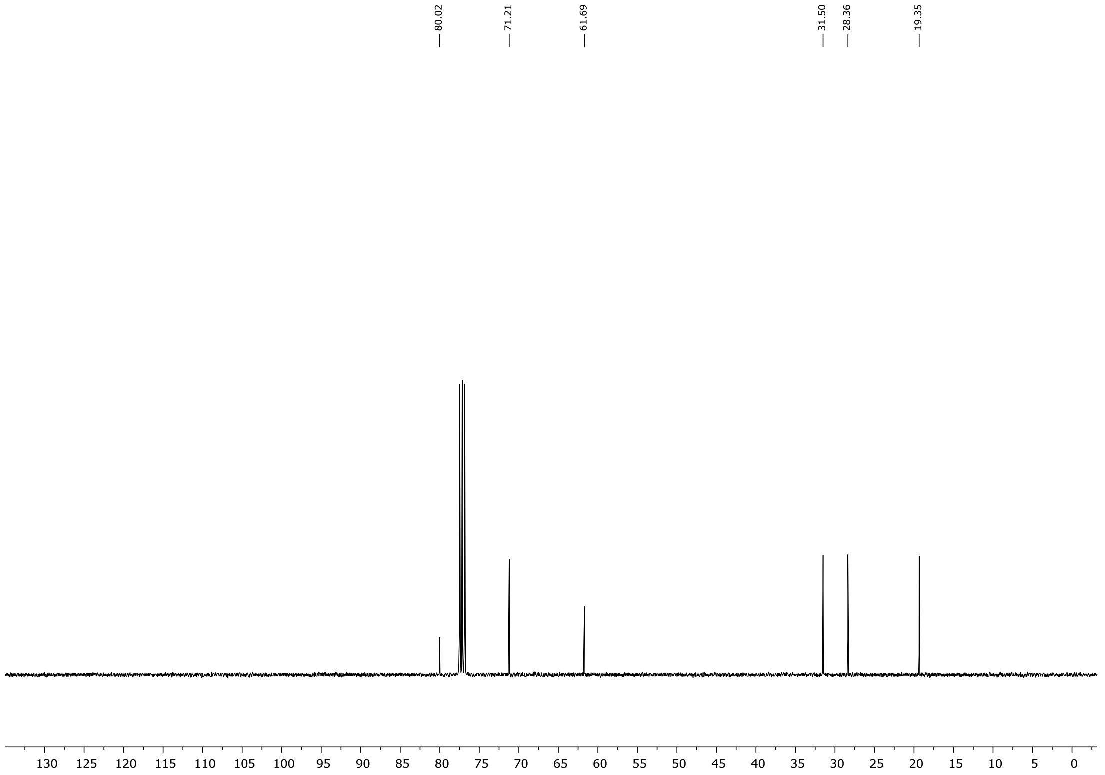

(3-((2-Nitrophenyl)selanyl)propyl)(prop-2-yn-1-yl)sulfane (6a)：

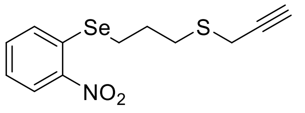

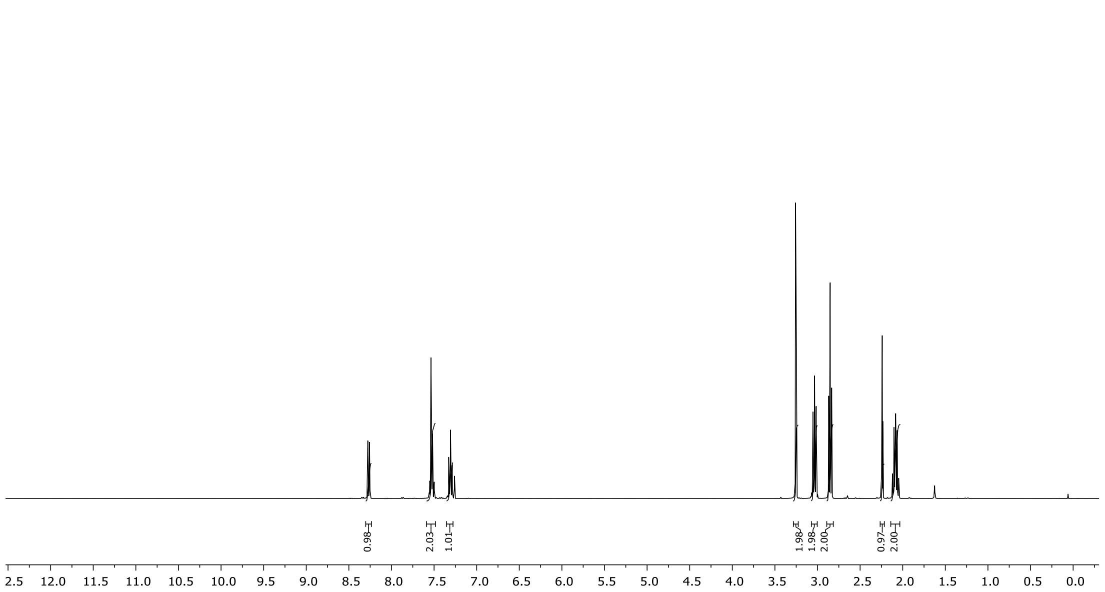

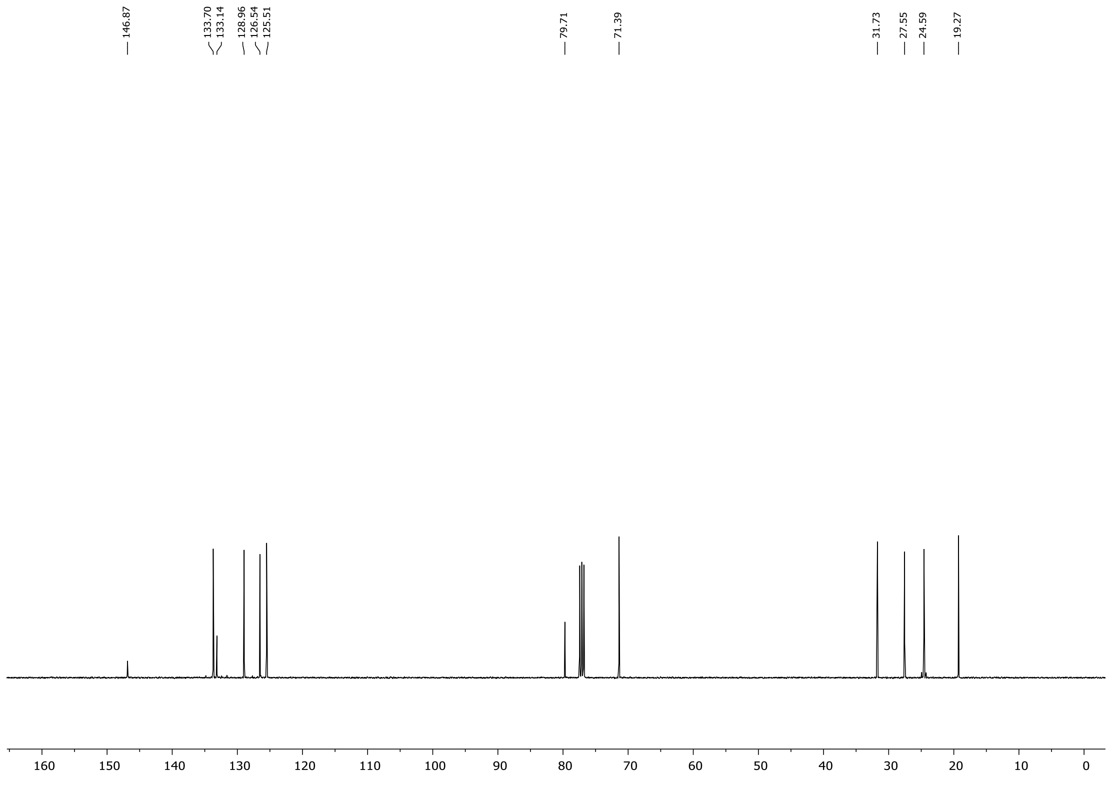

(3-(Phenylselanyl)propyl)(prop-2-yn-1-yl)sulfane (6b):

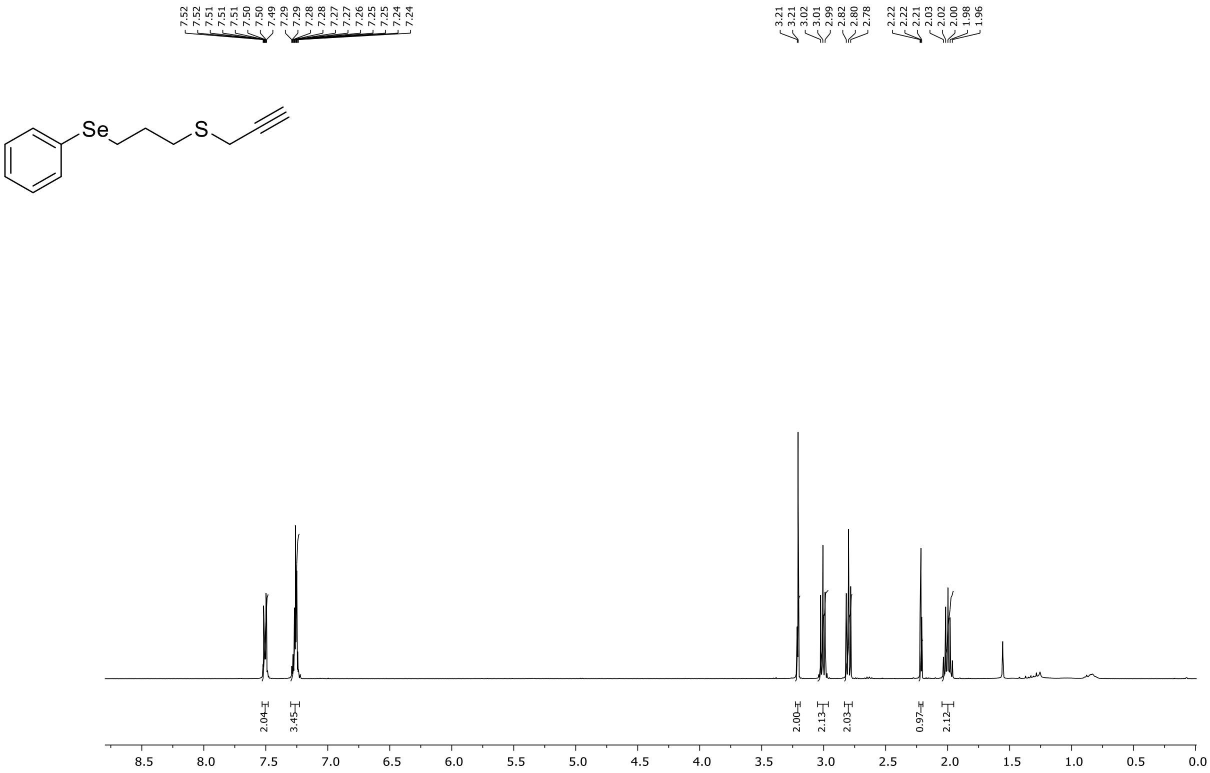

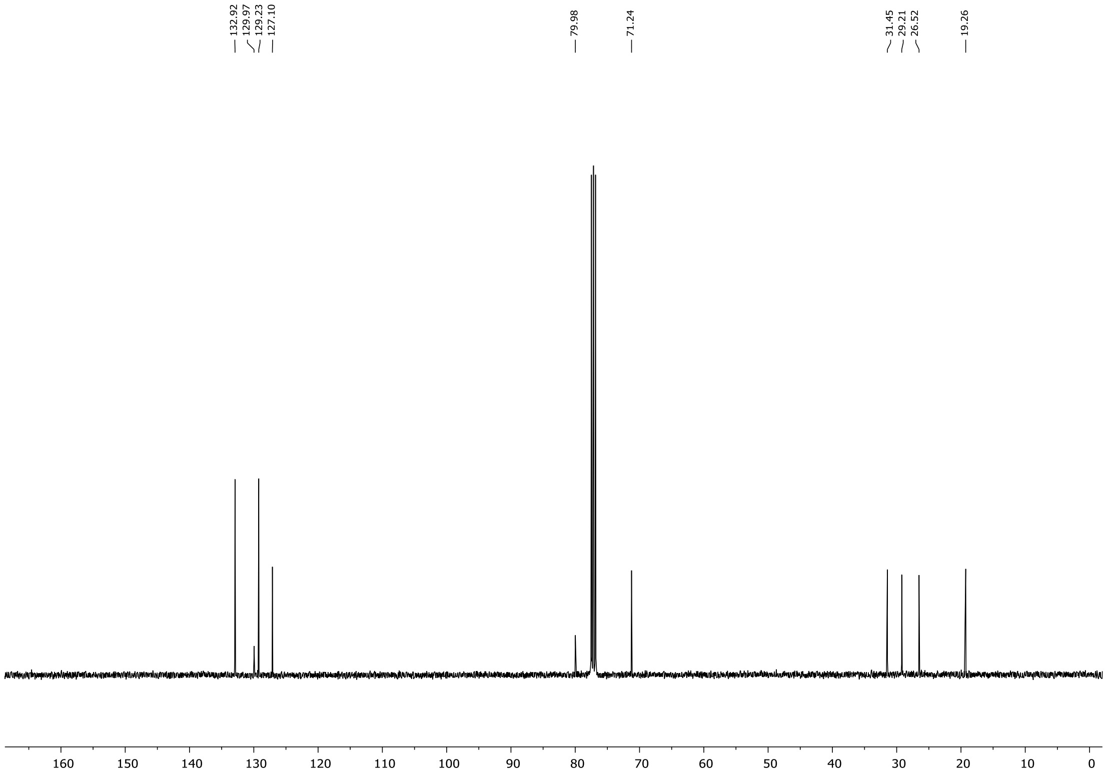

(*E/Z*)-(3-((3-((2-Nitrophenyl)selanyl)propyl)thio)prop-1-en-1-yl) ethanethioate (7a):

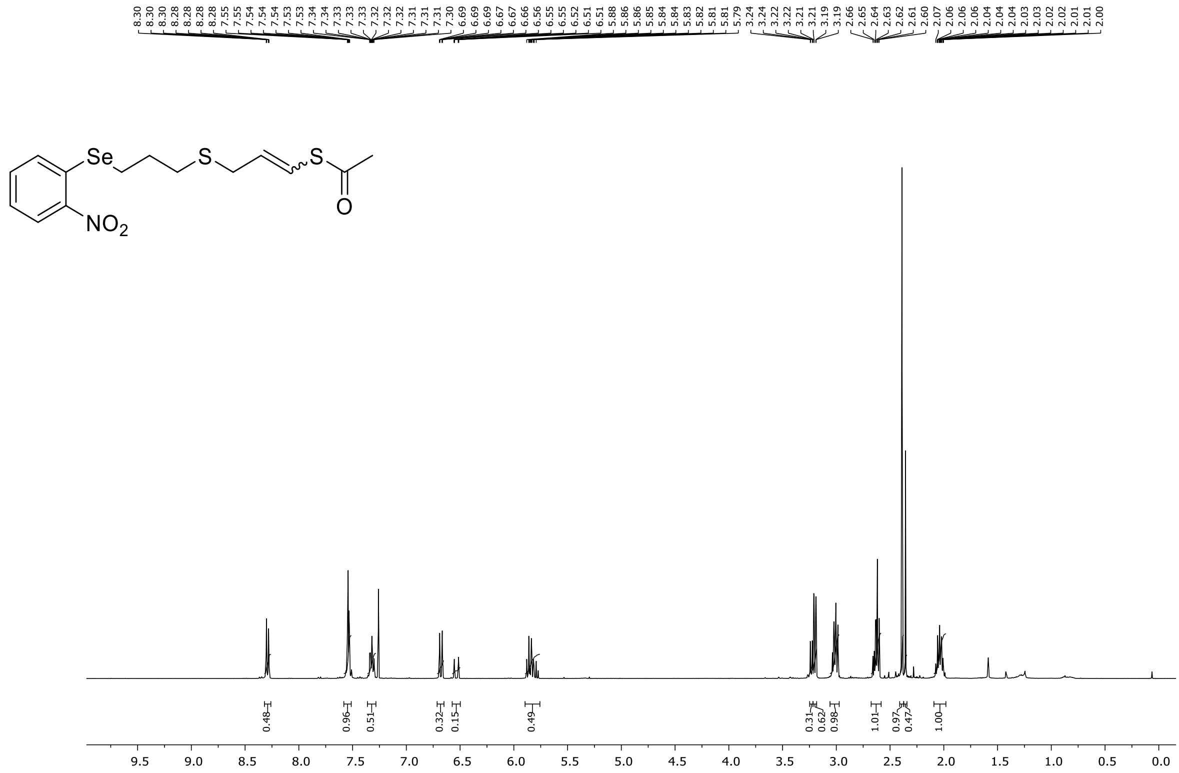

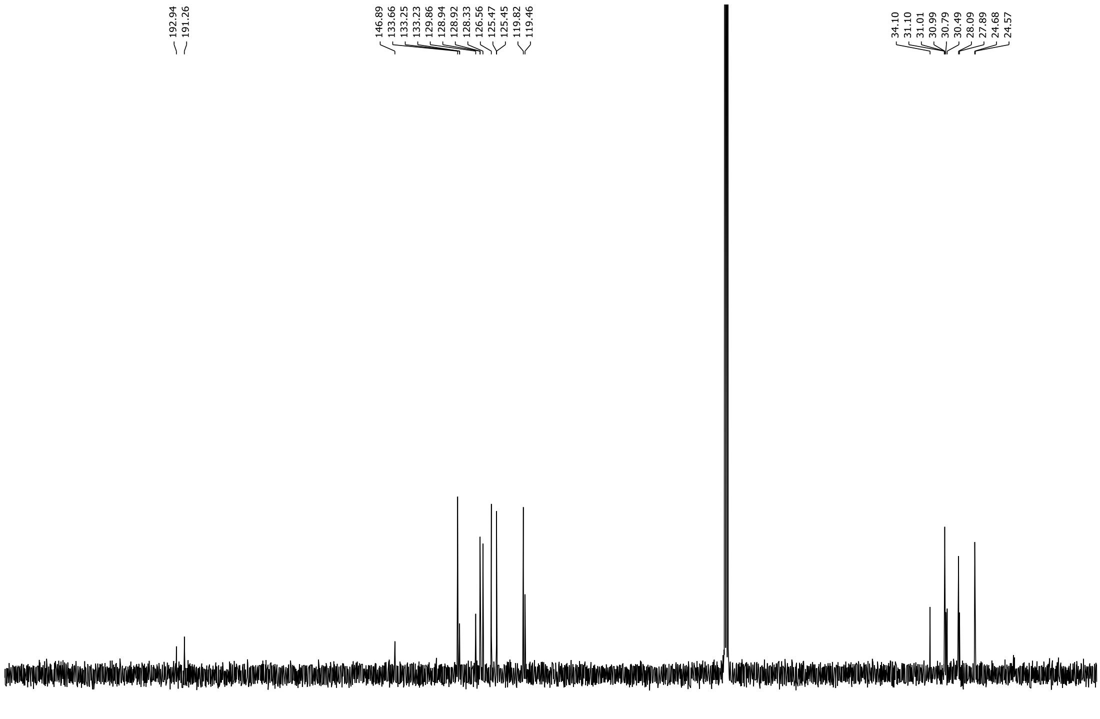

(*E/Z*)-3-((3-(Phenylselanyl)propyl)thio)prop-1-en-1-yl) ethanethioate (7b):

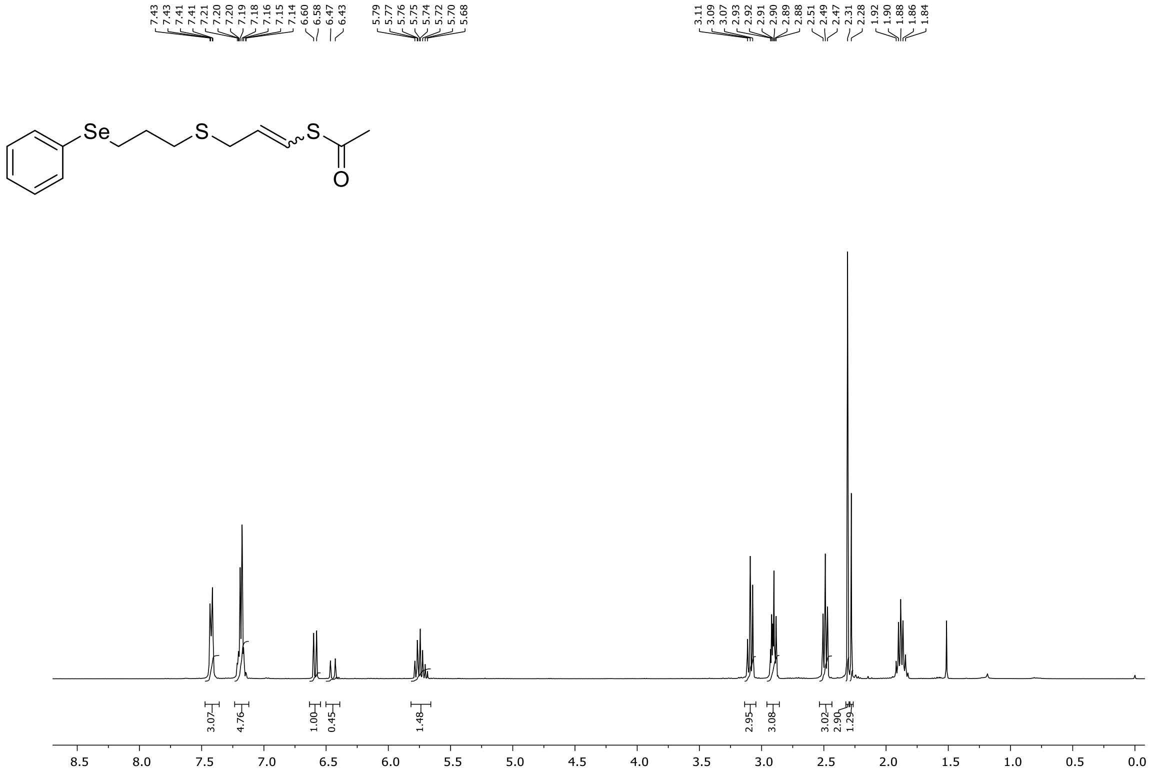

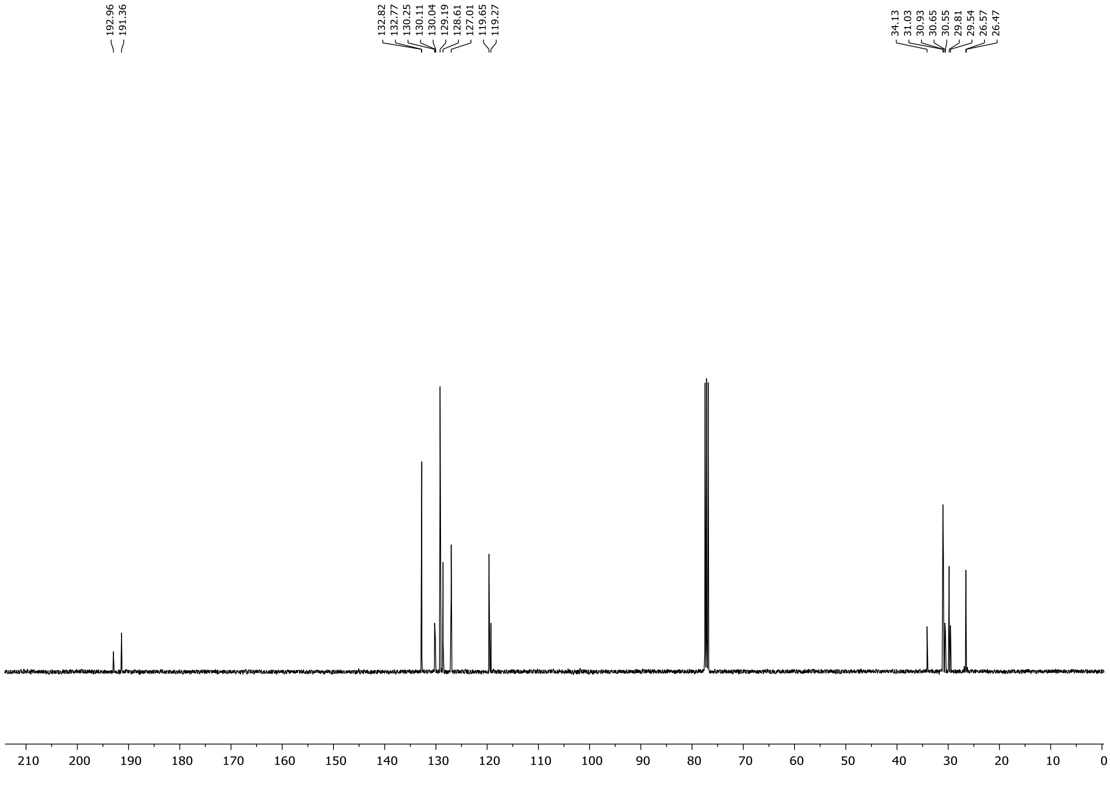

(*E/Z*)-1-Allyl-2-(3-((3-((2-nitrophenyl)selanyl)propyl)thio)prop-1-en-1-yl)disulfane (9a):

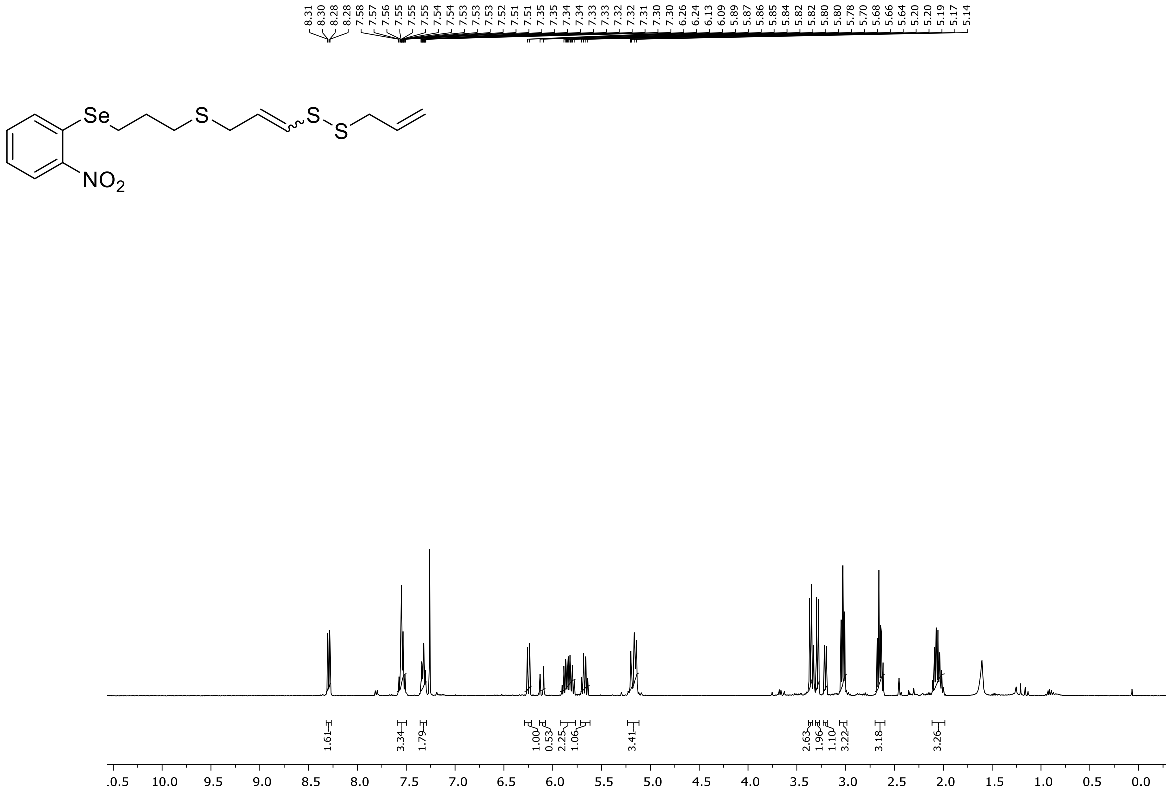

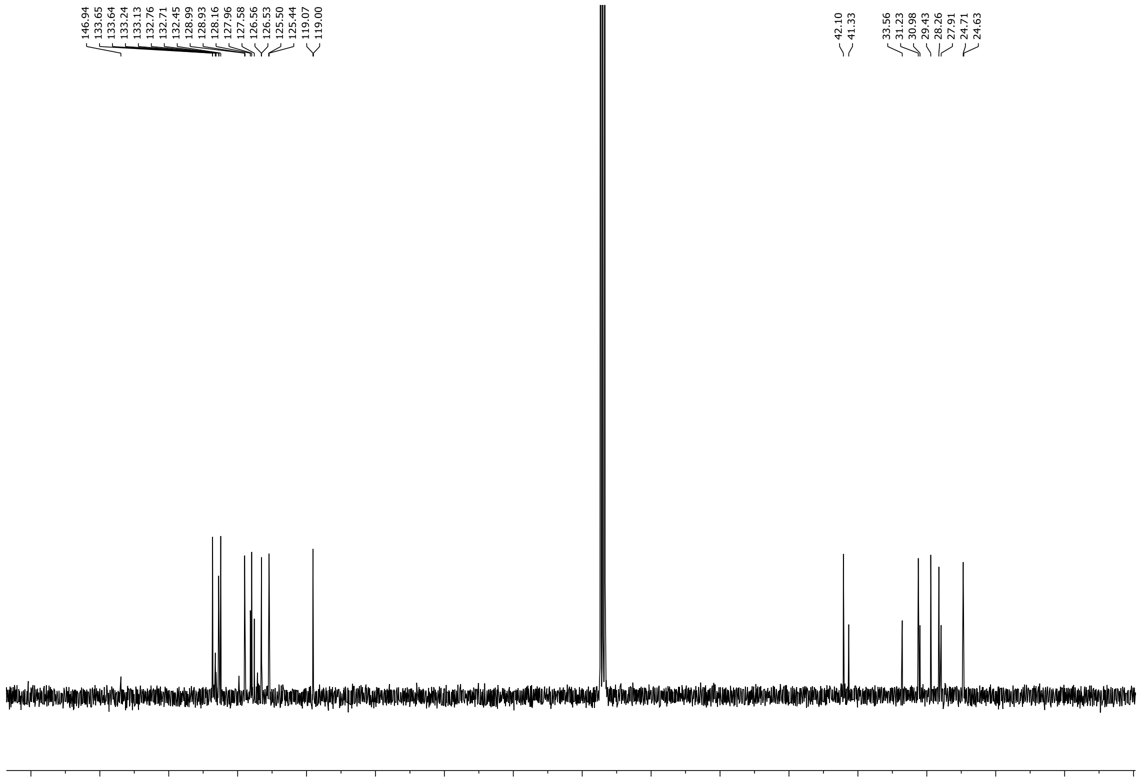

(*E/Z*)-1-Allyl-2-(3-((3-(phenylselanyl)propyl)thio)prop-1-en-1-yl)disulfane (9b):

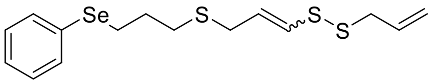

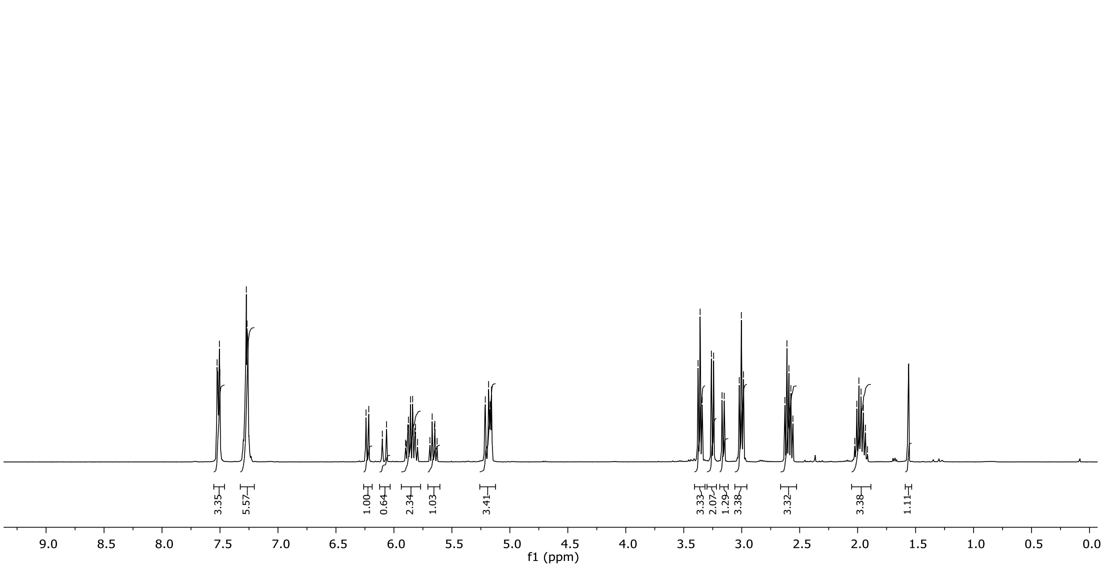

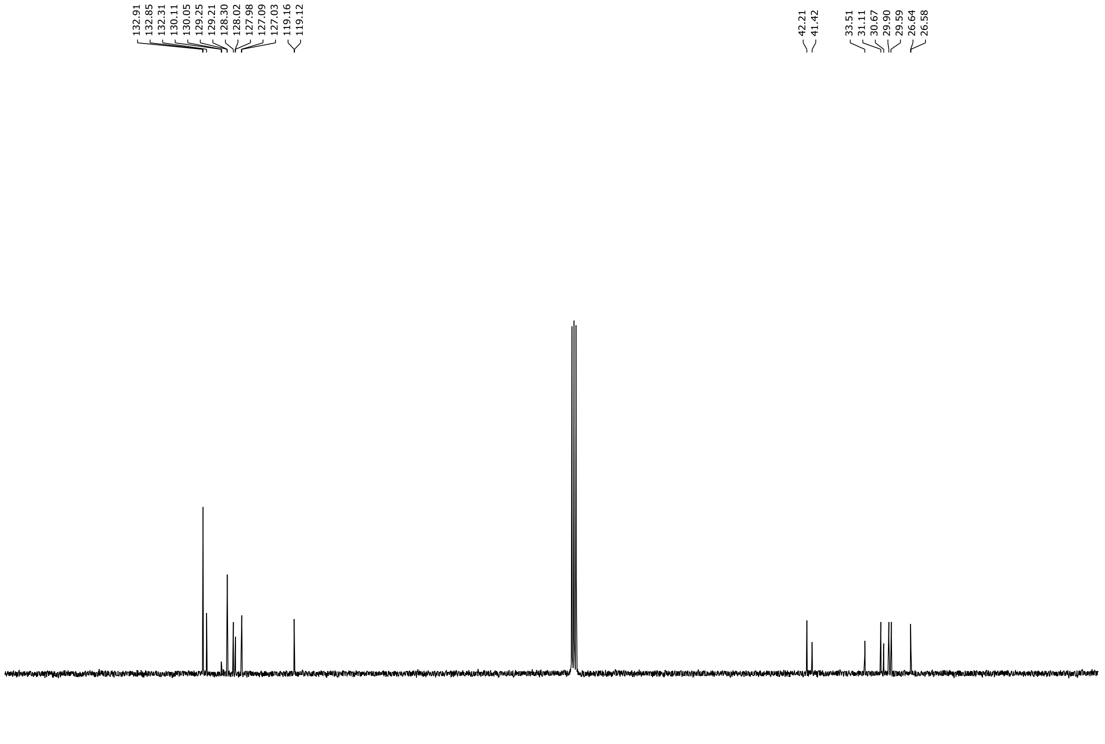

(*E*)-1-Allyl-2-(3-(allylsulfinyl)prop-1-en-1-yl)disulfane "Ajoene" (2):

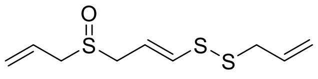

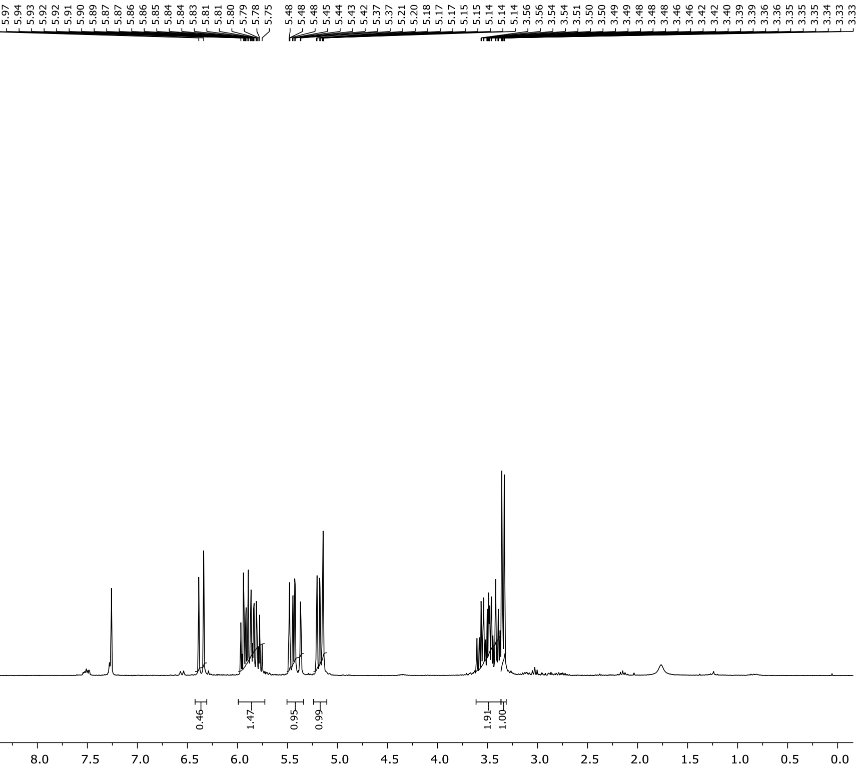

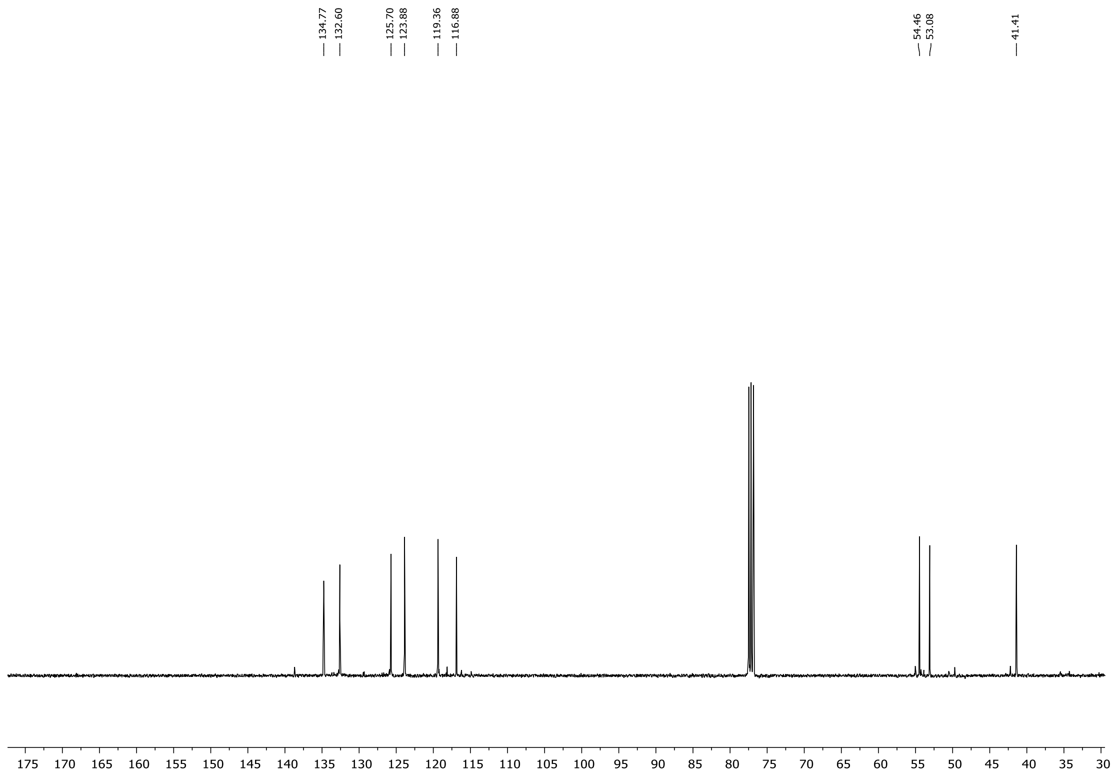

(*Z*)-1-Allyl-2-(3-(allylsulfinyl)prop-1-en-1-yl)disulfane “Ajoene” (2):

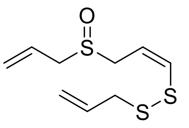

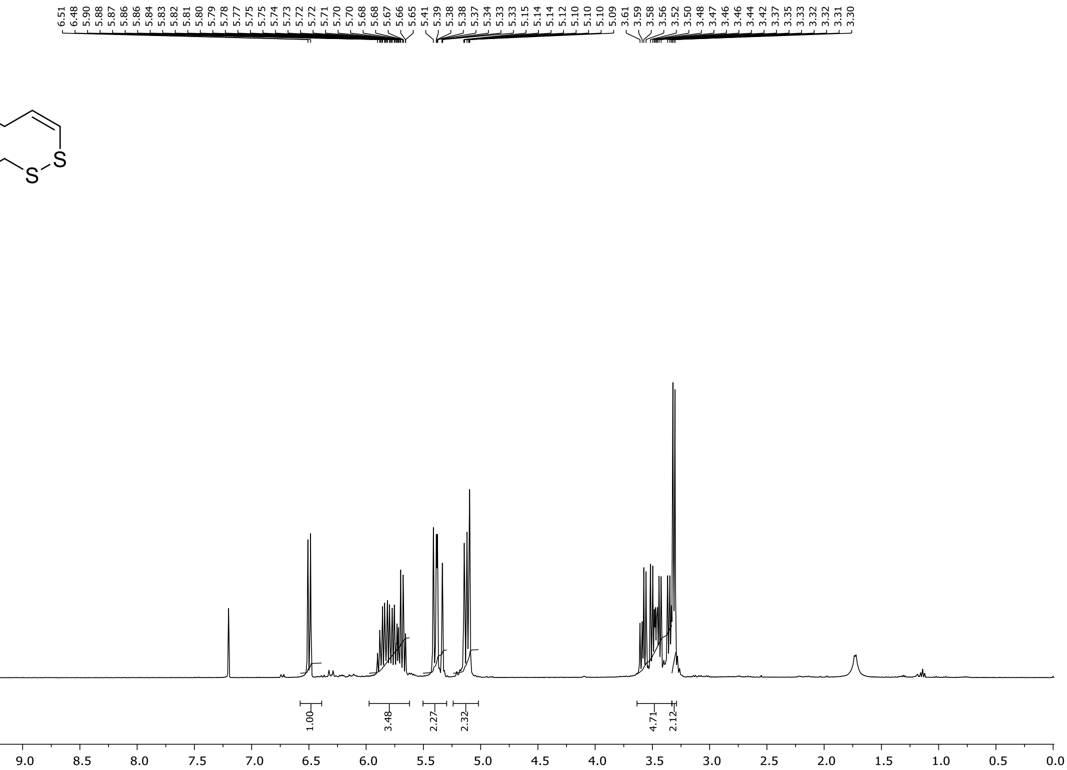

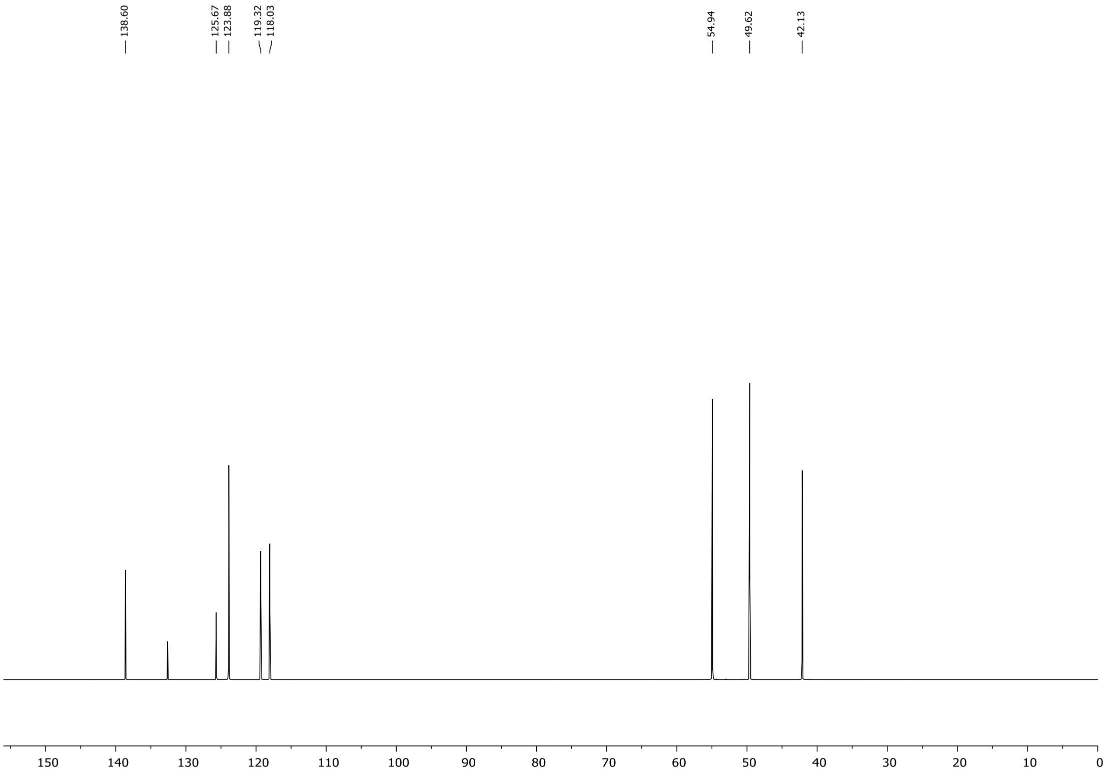

(*E/Z*)-S-(3-(Allylsulfinyl)prop-1-en-1-yl) ethanethioate (10):

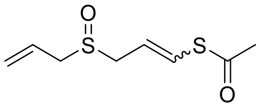

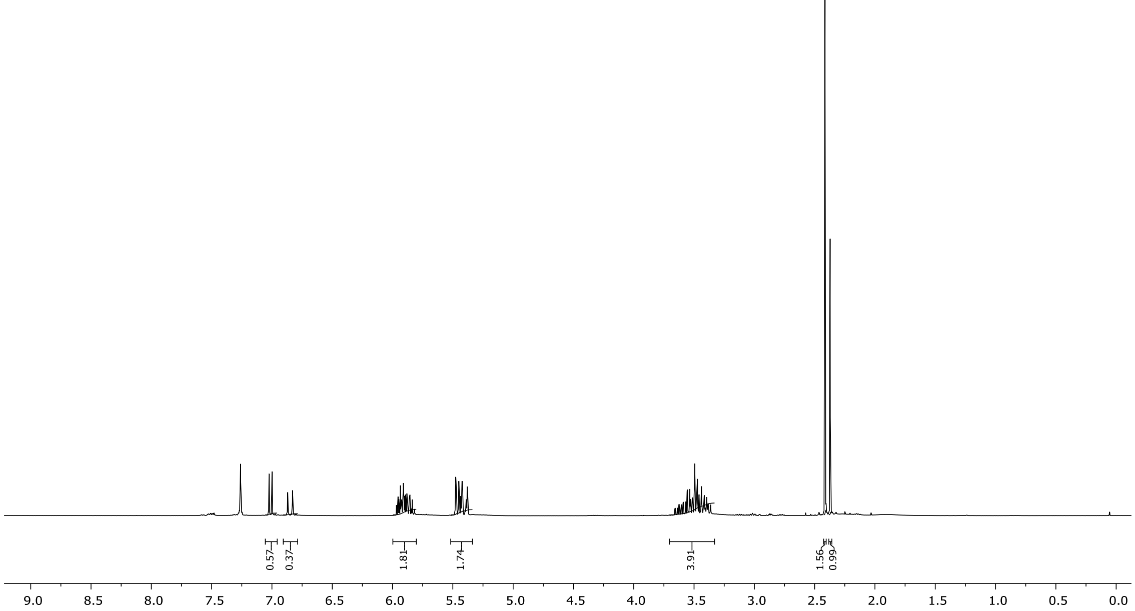

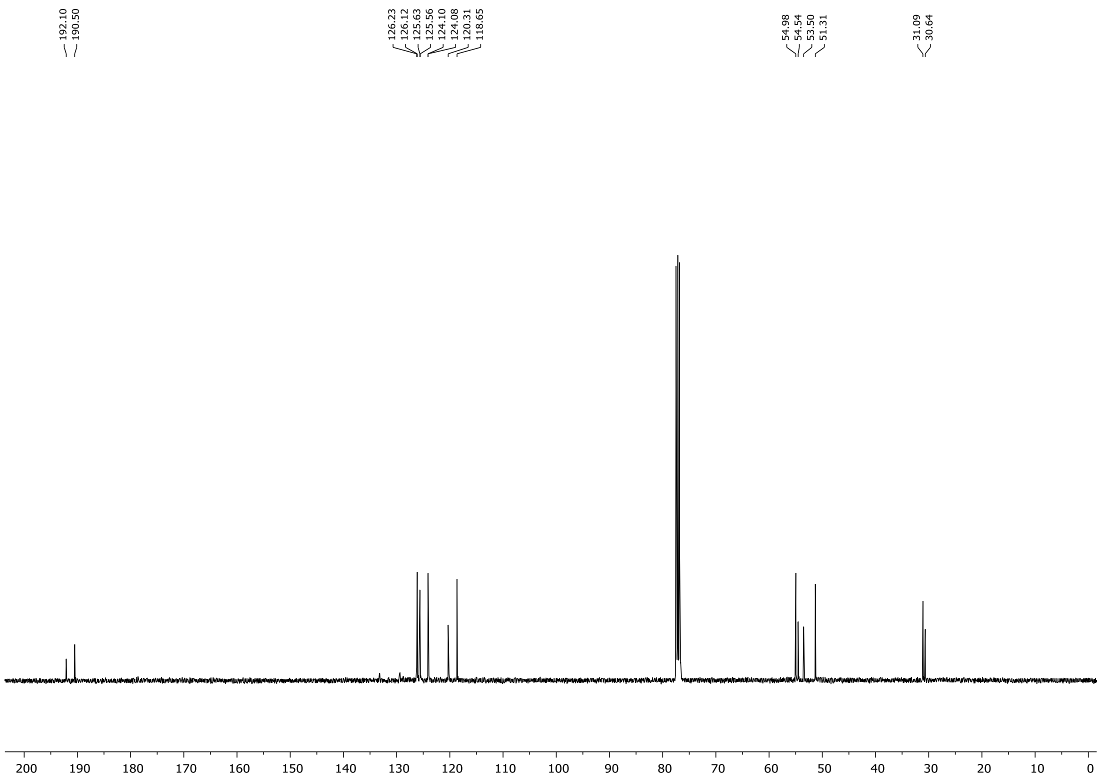

(*E/Z*)-S-(3-((3-(Phenylselanyl)propyl)sulfinyl)prop-1-en-1-yl) ethanethioate (11):

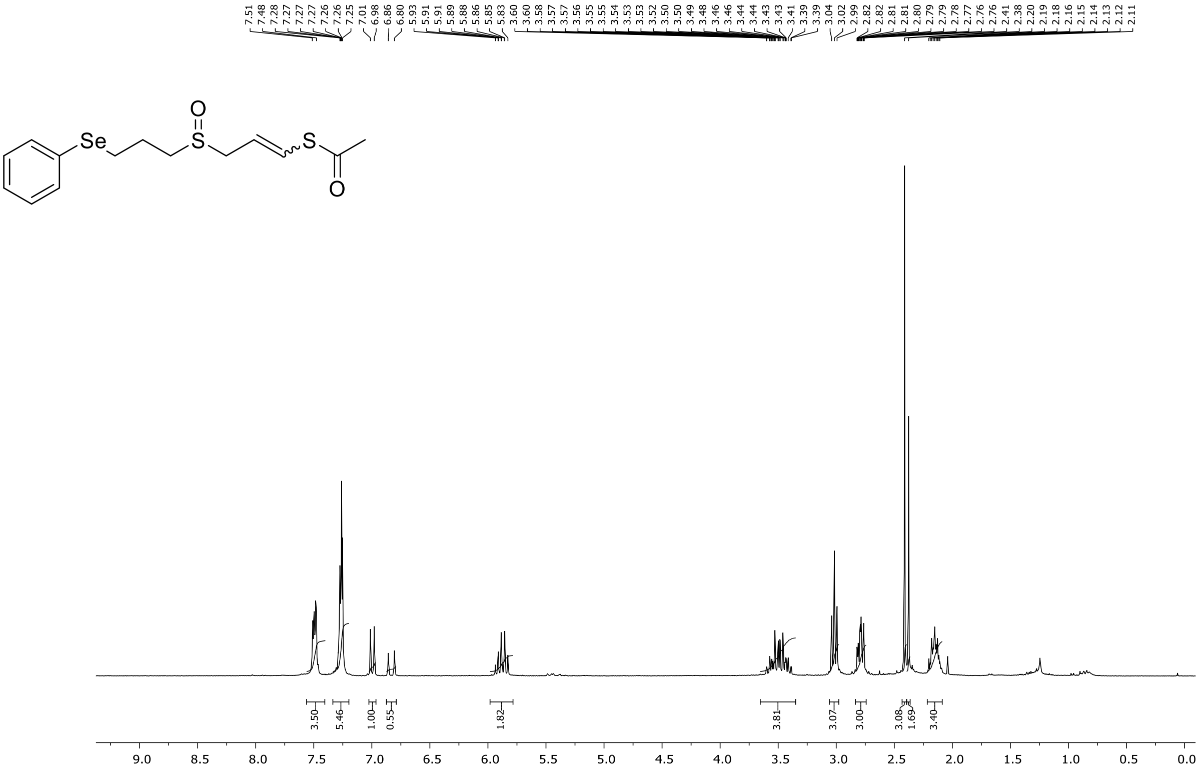

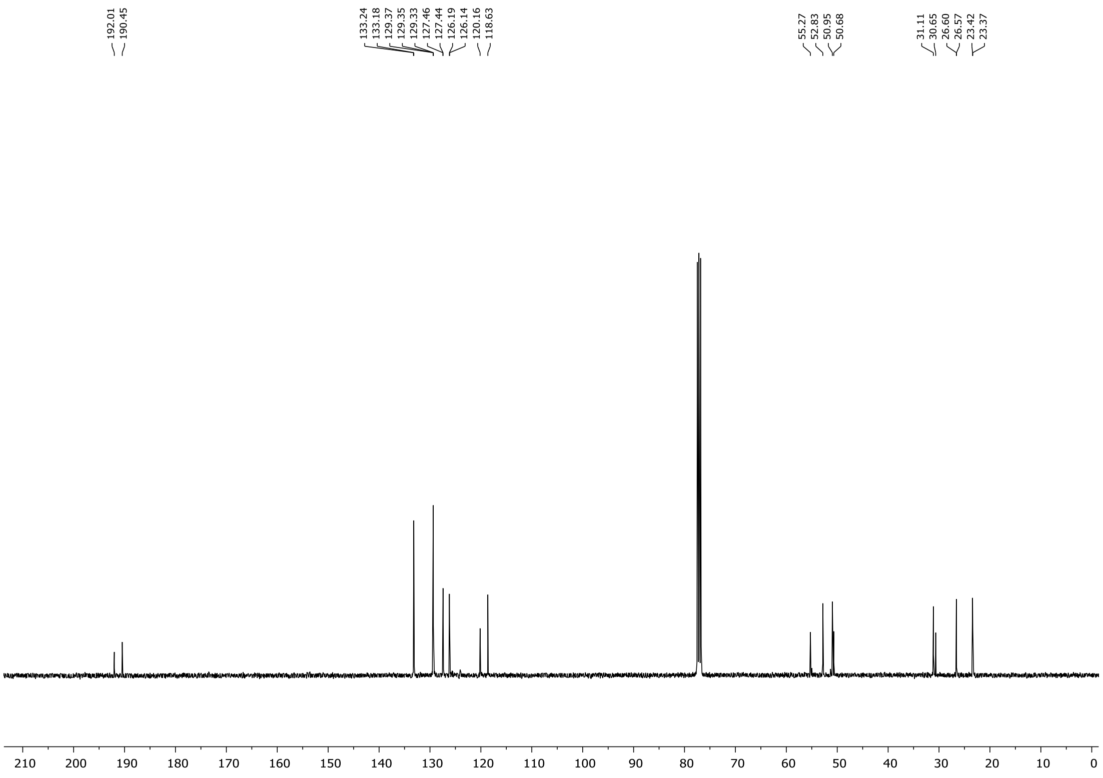

***S*-Allyl 4-methylbenzenesulfonothioate (8):**

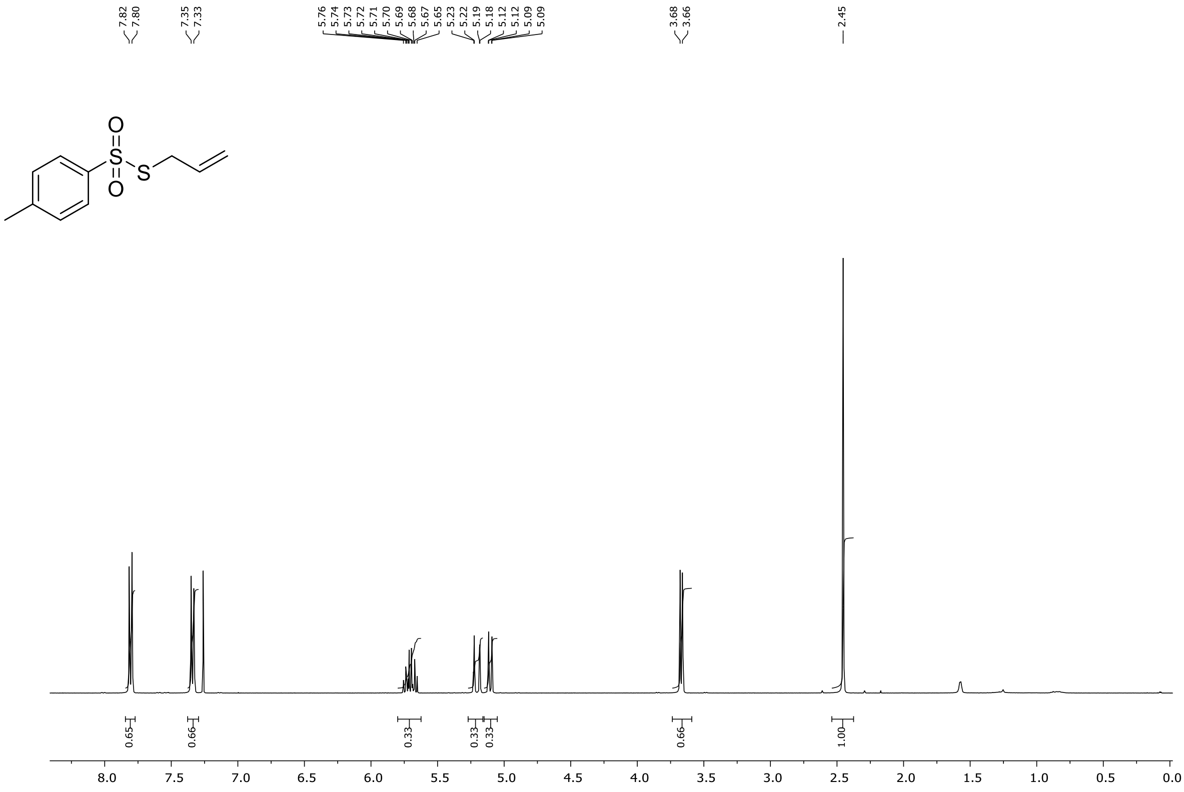

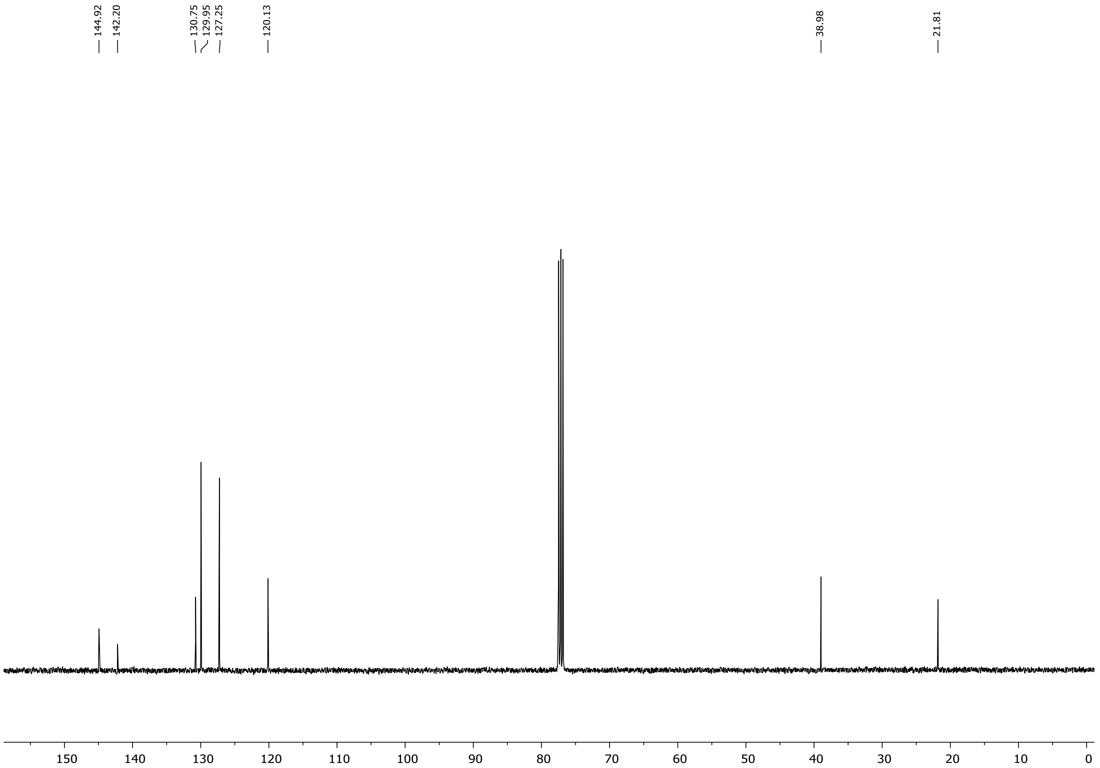

Supplement: Supplementary file 1 — Supplementary [file ANIE-57-12290-s001.pdf]
